# Architectural design adaptation of Egyptian residential buildings to accommodate digesters of biogas from food waste

**DOI:** 10.1038/s41598-025-90359-8

**Published:** 2025-03-11

**Authors:** Yasser O. El Gammal, Hamees M. El-Sheikh, Seleem S. E. Ahmad

**Affiliations:** 1 Department of Architectural Engineering, Faculty of Engineering, Zagazig University, P.O. Box 44519, Zagazig, Egypt; 2 Department of Material Engineering, Faculty of Engineering, Zagazig University, P.O. Box 44519, Zagazig, Egypt

**Keywords:** Sustainability, Biogas, Food waste, Architecture design renewable energy biogas plant prototypes, Environmental sciences, Energy science and technology, Engineering

## Abstract

The present work explores the relationship between architectural design and biogas production from household food waste and other disposable materials. It investigates the necessary parameters for adapting domestic architectural designs to accommodate biogas production plants. To achieve this, the study initially delves into commonly known biogas key parameters and selects the most relevant ones to be used as design guidelines for architects. It also examines the architectural structures of biogas digesters implemented worldwide to identify suitable prototypes that can be readapted or redesigned for Egyptian domestic environments. The paper also addressed some issues regarding required workspace calculations for the biogas roof installations, including bearing loads on roof slabs. The paper further explores the architectural characteristics of different types of Egyptian residential buildings to allow for the design of a domestic biogas plant prototype tailored to each specific building type. Additionally, it addresses the need to understand the architectural characteristics of Egyptian residential buildings and the design of the biogas plant prototype, highlights the constraints of Egyptian residential buildings to optimize the design, and proposes spatial configurations for biogas plants in various types of residential buildings in Egypt. Under the discussion section, the paper introduced some proposals regarding safety concerns and cost analysis.

## Introduction

### Background

This section introduces a wrap-up of the biogas concept, food waste, digesting processes, and commonly known biogas production key parameters and underscores the most relevant ones that might be useful as design guidelines for architects. Typically, biogas digesters vary in size and scale, depending on their biogas production capacity and unique design elements. While there is no universal prototype design for biogas digesters, except for three main types used in domestic biogas plants^1^: - Fixed Dome Digesters, Floating Cover/Drum Digesters, and Balloon/Tube Digesters – while these designs have emerged from diverse individual experiments worldwide, notably in Egypt, there isn’t a standardized biogas plant prototype tailored for different residential building types in the country.

#### Food waste

Lack of food resources in many places, climate change, depletion of fossil fuels, and increasing energy demand represent major challenges to today’s world. Both the World Bank and the United Nations encourage finding other sources of energy that are sustainable, clean, and renewable. Around one-third of food produced globally is estimated to be lost or wasted, or about 1.3 billion metric tons, resulting in economic losses of approximately 1 trillion US dollars^2,3^. However, the global biogas market is rapidly growing. Between 2022 and 2023, the global biogas market grew from 71.59 billion to $78.25 billion US, and it is expected to grow to 102.7 billion US by 2027^4^.

Food waste (FW) generation represents a complex and multifaceted issue that affects the three pillars of sustainability: economic, social, and environmental^5^. Nevertheless, the literature did not explore the potential impacts that FW from food services could have on sustainability pillars. Conventionally, food waste was regarded as the food losses (FL) accrued during the retail and final consumption phases; therefore, its generation is mainly attributable to the behaviors of retailers and consumers^6^. FW contributes to the emissions of greenhouse effect gases in storage, distribution, and transportation operations, landfill disposal because of methane emissions, and other disposal operations like incineration.

The increasing population growth worldwide leads to an increase in the generation of FW in huge amounts. FW consists of vegetable market waste, restaurant food waste, kitchen waste, etc. It is the main element of municipal solid waste (MSW)^7^. A promising renewable energy source is using food waste to produce biogas since it is a cheap and applicable process. Biogas is a smart energy source that is effective for numerous purposes. The production of biogas positively participates in the decentralization of energy generation at the national level.

**Fig. 1:**
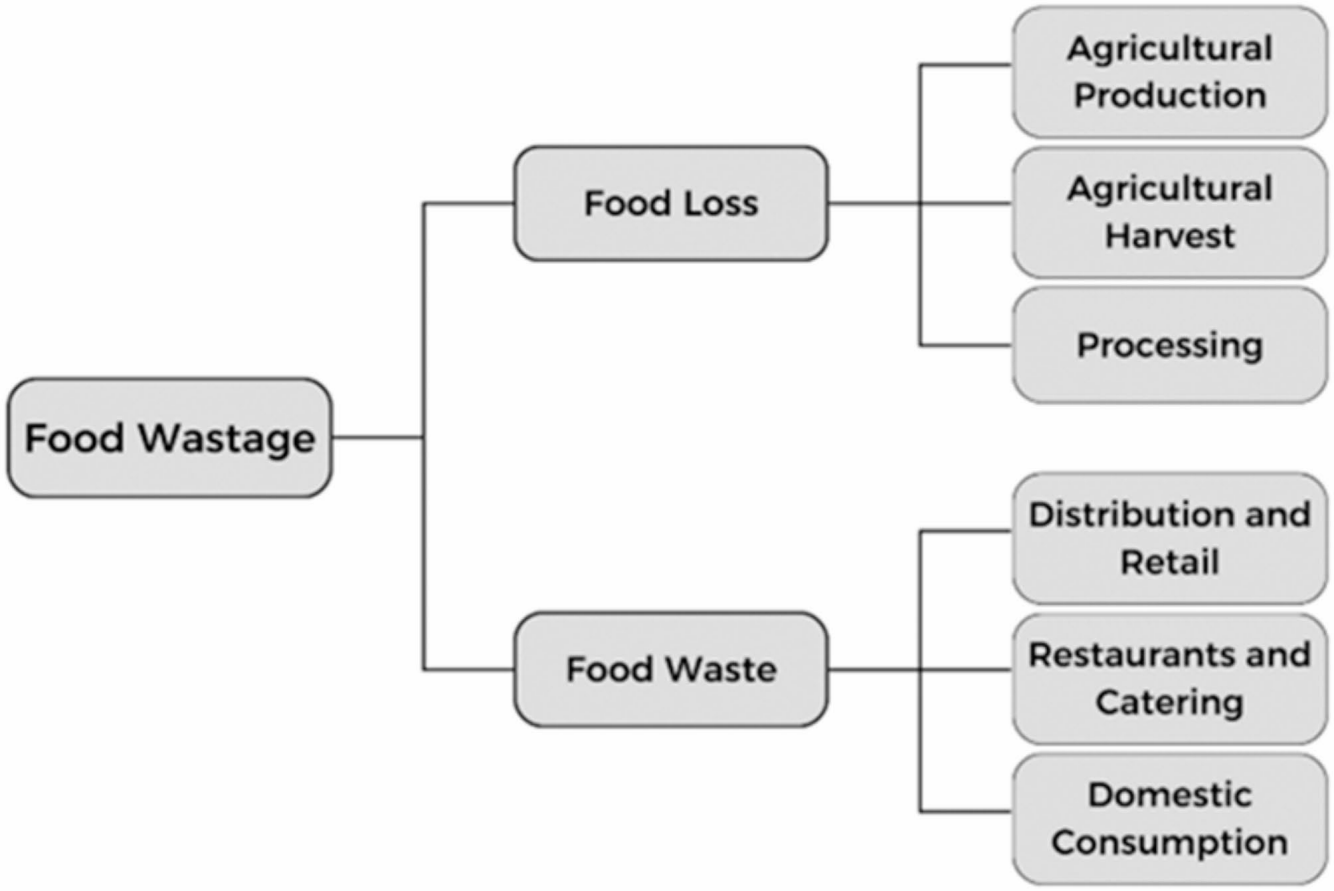
Food waste (FW) classification. Ref. [Author]

According to the FAO, food waste (FW) is the elimination of food from the food supply chain (FSC) that is edible but is either discarded or allowed to spoil due to negligence, primarily happening at the household level^8^. Despite the many definitions in the scientific literature for FW, achieving standardization is crucial for studying and understanding this topic^9^. Food waste is the best source for biogas production at a community level. Since it forms a rich nutritive environment for microbes that are needed for the fermentation process, leading to the production of biogas^10^. Its composition varies according to its components and types. It is also primarily composed of proteins, carbohydrates, lipids, and traces of inorganic compounds. Sources of FW in the literature, according to the European Commission in 2014, three categories of FW sources were classified based on the different stages of the food supply chain, as follows^11^. Inevitable food waste: It refers to nutrients lost during the eating phase (fruit pits, vegetable peelings, fruit peels, etc.). Unnecessary food waste: It is waste food that could have been eaten but was disposed of at the eating stage. Food losses: These are food commodities that are lost during their production. Food waste was also divided into five generation sources: agricultural production, agricultural harvest, processing, distribution, and consumption^12^, as presented in Fig. 1.

#### Biogas

Biogas is a sustainable gaseous energy source. It is generated from various raw materials like agricultural waste, manure, municipal waste, plant material, sewage, green waste, wastewater, and food waste. This production process involves digestion by organisms or methanogens within a digester (also called biodigester or bioreactor). As a versatile energy source, biogas finds applications as a fuel for heating and cooking. Moreover, it can be utilized in gas engines to transform the gas energy into both electricity and heat^13,14^.

#### Digesting processes

A “Digester” is a system that biologically digests organic waste material with the help of adding specific types of genetically treated micro-organisms to vegetable and fruit waste and other “Agro-food” biomass residuals to generate biogas^15^. There are two major types of biological digesters:

#### Aerobic digesters

Aerobic digestion occurs in the presence of oxygen and produces biogas. Organic material is oxidized, producing biogas and other chemical substances like nitrate, phosphate, and carbon dioxide^16^. Aerobic digestion is a fast process that runs at ambient temperature and is much less complex than anaerobic digestion^17^. Aerobic digestion: Is simple in construction, requires no cover for its tank, can be easily controlled, does not generate nuisance odors, generates low nutrient concentrations, and eliminates ammoniacal compounds^18^.

#### Anaerobic digesters

Anaerobic Digestion (AD) is a microbiological process that degrades organic waste without oxygen^19^. AD is an environmentally friendly technique extensively employed to address organic waste problems while producing renewable energy and achieving sustainable development. Studies and applications implementations of AD have grown notable in recent decades^20,21^, attributable to many reasons, particularly its suitability in treating any biodegradable residue, such as food waste or municipal waste, along with the need for the production of renewable energy and other waste disposal options that are divergent from landfilling^22^.

The composition and moisture content of FW makes it perfectly suitable for AD^23^. Generally, proteins and carbohydrates are rapidly transformed into a common biogas production. In contrast, lipids present slower biodegradability development; however, they offer better quality and level of biogas^20^. Consequently, comprehending the mechanisms of the AD process, key parameters, and all involved reactions is crucial.

### Commonly known biogas production key parameters

#### Chemical parameters

In general, the biogas composition is primarily methane (CH) and carbon dioxide ($\:{\text{C}\text{O}}_{2}$) and may have small amounts of hydrogen sulfide ($\:{\text{H}}_{2}\text{S}$), moisture, and siloxanes. The high amounts of FW might result from deficient storage facilities, packaging, infrastructure, and insufficient market facilities^24^. FW contains a high content of nitrogen, potassium, carbon, phosphorus, and other elements^25^. The C/N ratio is a significant parameter, measuring FW’s potential for energy recovery^26^. FW composition: It contains 15–25% proteins, 13–30% lipids, and 41–62% degradable carbohydrates, with a high VS fraction of approximately 85% ± 5%, 74–90% high moisture content, and 5.1 ± 0.7 mean acid PH^27^.

The initial part of FW generation (livestock and agriculture) mainly consists of non-edible substances that have been separated from the feedstocks. The composition of food waste generated during the final stage of the food supply chain (markets) differs from that of earlier stages, where additional material fractions are included, like paper, glass, plastics, metals, etc., which originate from packaging^28,29^.

The composition of FW consumer’s produce closely depends on diverse cooking and eating habits. Due to these reasons, numerous researchers have characterized FW, each delineating its components and characteristics based on its geographical location, local products, social behaviors, origin, habits, etc^30^. Biogas produced from Anaerobic Digestion usually consists of Methane. $\:\text{C}{\text{H}}_{4}$, Ammonia $\:{\text{N}\text{H}}_{3}$, Water $\:{\text{H}}_{2}\text{O}$, Oxygen O, Nitrogen N, Carbon dioxide $\:{\text{C}\text{O}}_{2}$, Hydrogen Sulphide $\:{\text{H}}_{2}\text{S}$, Hydrogen $\:{\text{H}}_{2}$, And other trace contaminants^31^.

The AD process serves as a way of recycling, recovering energy, and utilizing it industrially as fertilizer, as well as a system that reduces landfill disposal, either partially by eliminating digestate or reusing it completely^22,32^. During the AD process, a series of metabolic reactions occur that follow the conversion of organic matter (OM) into methane and carbon dioxide, as well as inorganic nutrients and compost^19^.

**Fig. 2:**
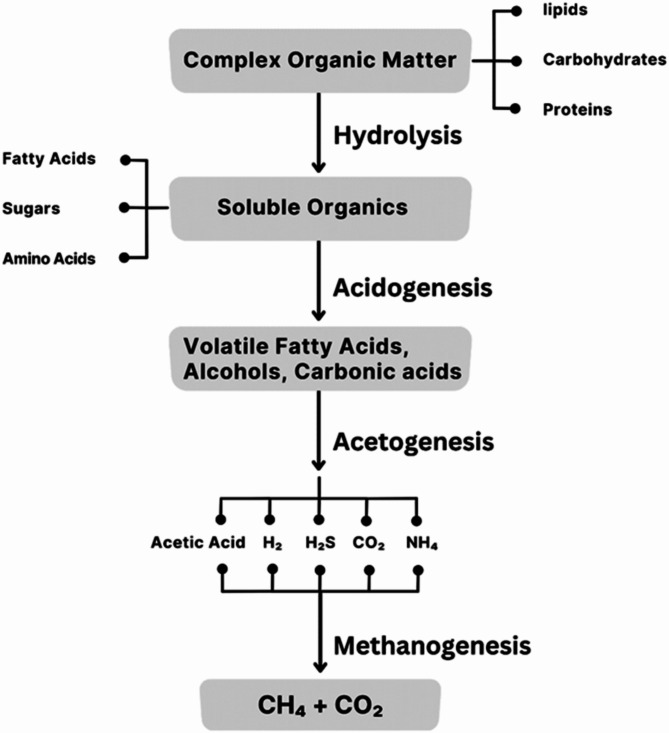
Stages of the AD Process. Ref. [Author]

As a result of this, organic matter is decomposed by anaerobic microorganisms under anaerobic conditions, resulting in an energy-dense biogas; this reaction happens simultaneously through four major intricate biochemical stages, namely, hydrolysis, acidogenesis, acetogenesis, and methanogenesis^33^, as shown in Fig. 2.

#### Physical parameters

The following is a summary of the physical parameters of biogas, specifically for its components, methane (CH₄) and carbon dioxide (CO₂)^34^:

*Methane (CH₄)*:

- Molecular Weight: The molecular weight of methane is 16.04 g/mol, reflecting its chemical structure and atomic composition.
- Proportion: Methane comprises 0.554 of the biogas mixture, making it the primary constituent of biogas.
- Boiling Point: The boiling point of methane is -164 °C, which means it is typically in gaseous form at standard temperatures.
- Freezing Point: The freezing point of methane is -182.5 °C, indicating that it remains in a gaseous state under normal conditions.
- Density: The density of methane is 0.66 kg/m2, which is lower than that of air, contributing to its tendency to rise in the atmosphere.
- Dangerous Temperature: The dangerous temperature threshold for methane is 64.5 °C, above which it can pose significant risks of combustion or explosion.
- Dangerous Pressure: Methane reaches a dangerous pressure point at 45.8 kg/cm2, indicating the pressure level at which it may become hazardous.
- Specific Heat Capacity: The specific heat capacity of methane at 1 kg/cm2 is 6.962 × 10^-4^/kg °C, reflecting the energy required to change the temperature of methane.
- Rate Cv/Cp: The ratio of specific heat at constant volume (Cv) to specific heat at constant pressure (Cp) for methane is 1.037, important for understanding its thermal behavior.
- Burning Heat: The burning heat of methane is 55,403 J/kg, indicating the amount of energy released during its combustion.
- Fire Propagation Rate: The rate of fire propagation in methane when mixed with air is 0.0581 (mass), highlighting its flammability and potential risk in confined spaces.

*Carbon Dioxide (CO₂)*:

- Molecular Weight: The molecular weight of carbon dioxide is 44.01 g/mol due to its carbon and oxygen composition.
- Proportion: CO₂ represents 1.52 of the biogas mixture, a significant but secondary component compared to methane.
- Boiling Point: The boiling point of carbon dioxide is -78.5 °C, which means it sublimes from a solid to a gas under standard conditions.
- Freezing Point: CO₂ freezes at -56.6 °C, changing from a gaseous state to a solid form.
- Density: Carbon dioxide is 1.82 kg/m2, making it heavier than air, which causes it to settle in low-lying areas when released.
- Dangerous Temperature: The dangerous temperature for CO2 is 48.9 °C, above which the gas may cause health risks, including suffocation in high concentrations.
- Dangerous Pressure: The dangerous pressure for CO2 is 73 kg/cm2, beyond which the gas may become hazardous due to the increased risk of liquefaction and the potential for explosion under confined conditions.
- Rate Cv/Cp: The Cv/Cp ratio for CO₂ is 1.303, which is slightly higher than methane, indicating a different thermal expansion behavior.

In summary, the physical properties of methane and carbon dioxide, including their molecular weight, boiling and freezing points, density, and dangerous conditions, are essential for understanding their behavior in biogas production, storage, and utilization. Methane is the primary combustible component, while carbon dioxide, although inert, plays a significant role in the overall composition of biogas.

#### Safety parameters

The most probable hazard scenarios for a biogas plant failure involve either the leakage of gas and residual water generated during the fermentation process within the plant or the occurrence of both leakages simultaneously. Gas leaks typically result from inadequately installed gas valves and pipes connected to the fermentation tank. On the other hand, water leakage may occur due to insufficient insulation or the use of inappropriate materials lining the internal walls of the biogas plant, potentially causing chemical reactions with the internal wall materials and resulting in erosion and, consequently, the formation of holes in the walls of the plant^35–37^. Underscoring the most relevant biogas production key parameters that might be useful as design guidelines for architects. Based on examining the essential Biogas Production Key Parameters outlined above, architects should focus primarily on understanding the physical and safety aspects of biogas when tailoring biogas plant designs for domestic and residential settings.

### Green/sustainable/environmental architecture

The concept of green architecture holds significant sway within contemporary architectural discourse, propelled by a worldwide push for sustainable community development. However, a pervasive conflation exists in the literature between sustainable, environmental, and green architecture, often interchangeably used to describe structures with comparable characteristics. Despite this ambiguity, green architecture potentially possesses a distinct advantage over the other two terms, represented by the fact that sustainability and environmental impact are included under the umbrella of green architecture and not the opposite.

Green buildings are pivotal in mitigating adverse impacts on the natural environment by conserving water, energy, and other resources, incorporating renewable energy sources and eco-friendly materials, recycling waste, and minimizing emissions. They can yield net positive effects, such as generating energy or fostering biodiversity. Among sectors contributing significantly to greenhouse gas emissions, the building industry stands out for its capacity to effect substantial reductions.

Adopting green building practices, which leads to these performance advantages, also translates into economic gains for various stakeholders. Developers reap rewards from increased property values resulting from efficient resource utilization and constructing durable, high-performing structures. Such buildings attract business owners and occupants due to their environmental friendliness, enhanced comfort, heightened efficiency, reduced waste, and lower operational expenses—factors that positively influence occupancy rates^38^.

### Architecture of biogas digesters

Generally, biogas digesters come in various sizes and scales, depending on the volume of biogas they can produce and the specific components of their design. However, according to existing literature, the three common domestic biogas plants are Fixed Dome Digesters, Floating Cover/Drum Digesters, and Balloon/Tube Digesters^1^.

#### Fixed dome digesters

**Fig. 3:**
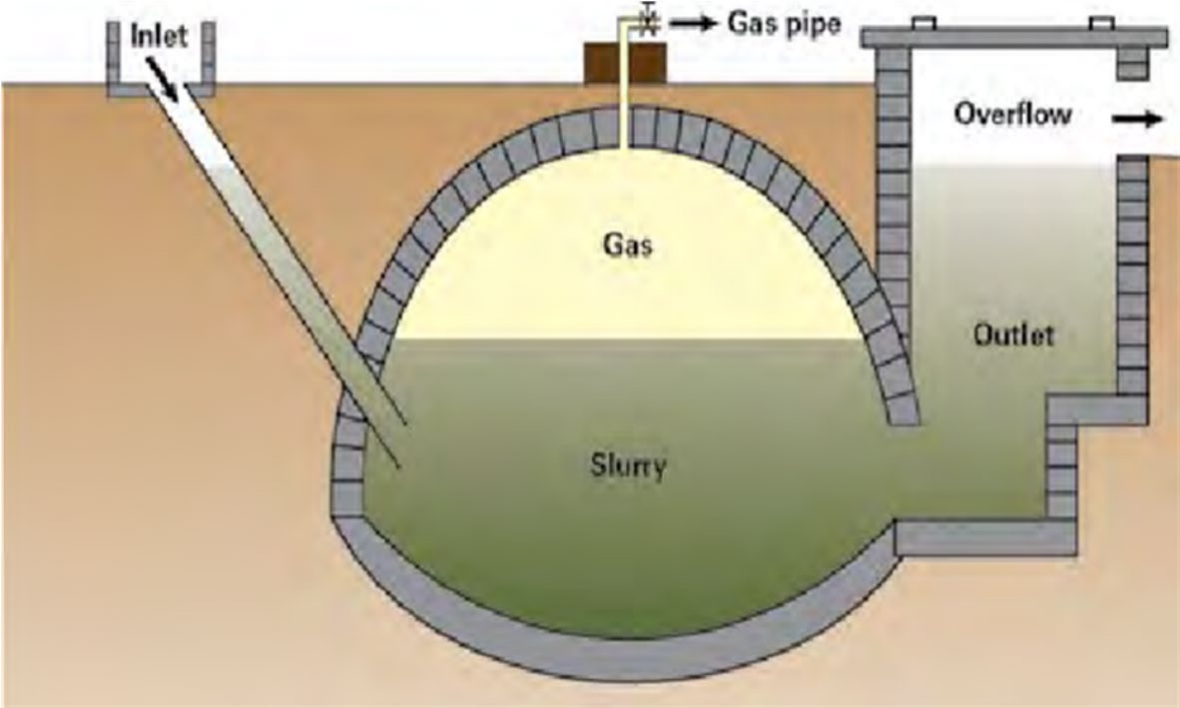
The general concept of the “Fixed Dome Digester”. Ref.:^39^.

A “Fixed-dome biogas plant”, Fig. 3, consists of a sealed dome-shaped digester housing an immovable, rigid gasholder, a feedstock inlet, and a displacement pit known as the “Compensation tank”. Gas generated in the digester is stored in the upper section of the reactor. As gas production increases, the pressure inside the digester rises, pushing the digestate into the compensation tank through a closed outlet gas valve. When the gas valve opens for utilization, the pressure drops, allowing a proportional amount of slurry to flow back from the compensation tank into the digester. This design results in continuous gas pressure variation based on production and use. Typically constructed underground, fixed-dome plants are shielded from low temperatures, with surrounding soil counteracting internal pressure (Usually 0.1–0.15 bar)^39^.

These plants are recommended only when experienced biogas technicians are available for construction, ensuring a gas-tight structure. Despite their modest initial cost and long operational lifespan (Approximately 15–20 years), fixed-dome plants may develop porosity and cracking over time, leading to gas leaks. While special sealants can address porosity, cracking often causes irreparable leaks. The fluctuating gas pressure in fixed-dome digesters may complicate gas utilization. Various designs exist, including the “Chinese fixed-dome plant,” the “Indian Dee Bandhu”, and the “Camartec model from Tanzania”. Fixed-dome digesters come in different sizes, typically ranging from 6 to 16 m^3^, with consistent design elements across all variations.

#### Floating cover/drum digesters

**Fig. 4:**
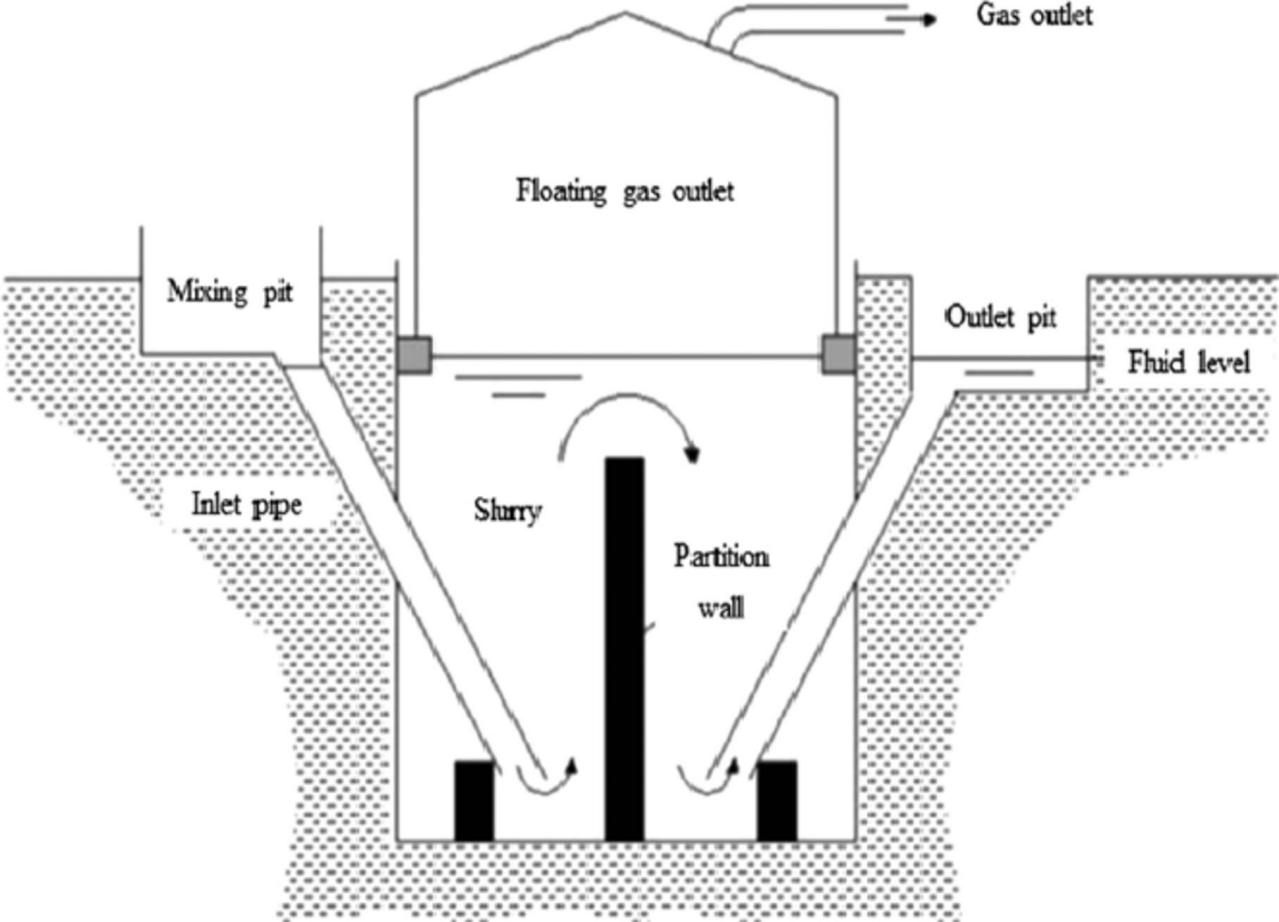
The general concept of the “Floating Cover Digester”. Ref.:^40^.

The “Floating drum digester “, Fig. 4, is typically constructed using concrete and steel, whereas the fixed dome digester is commonly built with locally available materials such as bricks and stones. In both types of digesters, only the cover of the floating drum digester is situated above ground, while the remaining components are housed underground, with a retention time of 20–30 days.

Floating drum digesters feature a steel cover that floats on the slurry, moving vertically to accommodate constant biogas pressure. Additional weight can be added to the top of the cover to increase gas pressure. Conversely, gas volume remains relatively constant in fixed dome digesters while pressure fluctuates. Despite these differences, the operational principles of both designs are similar. Feedstock is introduced directly or after mixing in a pit through an inlet pipe into the digester tank. Biogas produced is collected above the slurry and exits the tank through a gas pipe connected to the top of the digester. The digested slurry is expelled through an outlet pipe into either an outlet pit or a displacement tank.

Depending on the configuration, the digester tank may have one or two compartments. In developing countries, the system lacks proper mixing and operates without temperature control. Additionally, there is no provision for removing settled inert materials, gradually reducing the digester’s effective volume over time. While the absence of moving parts and simple construction make this digester easy to operate and maintain, the high installation cost and requirement for skilled craftsmen limit the widespread adoption of this technology in developing countries^40–42^.

#### Balloon/tube digesters

**Fig. 5:**
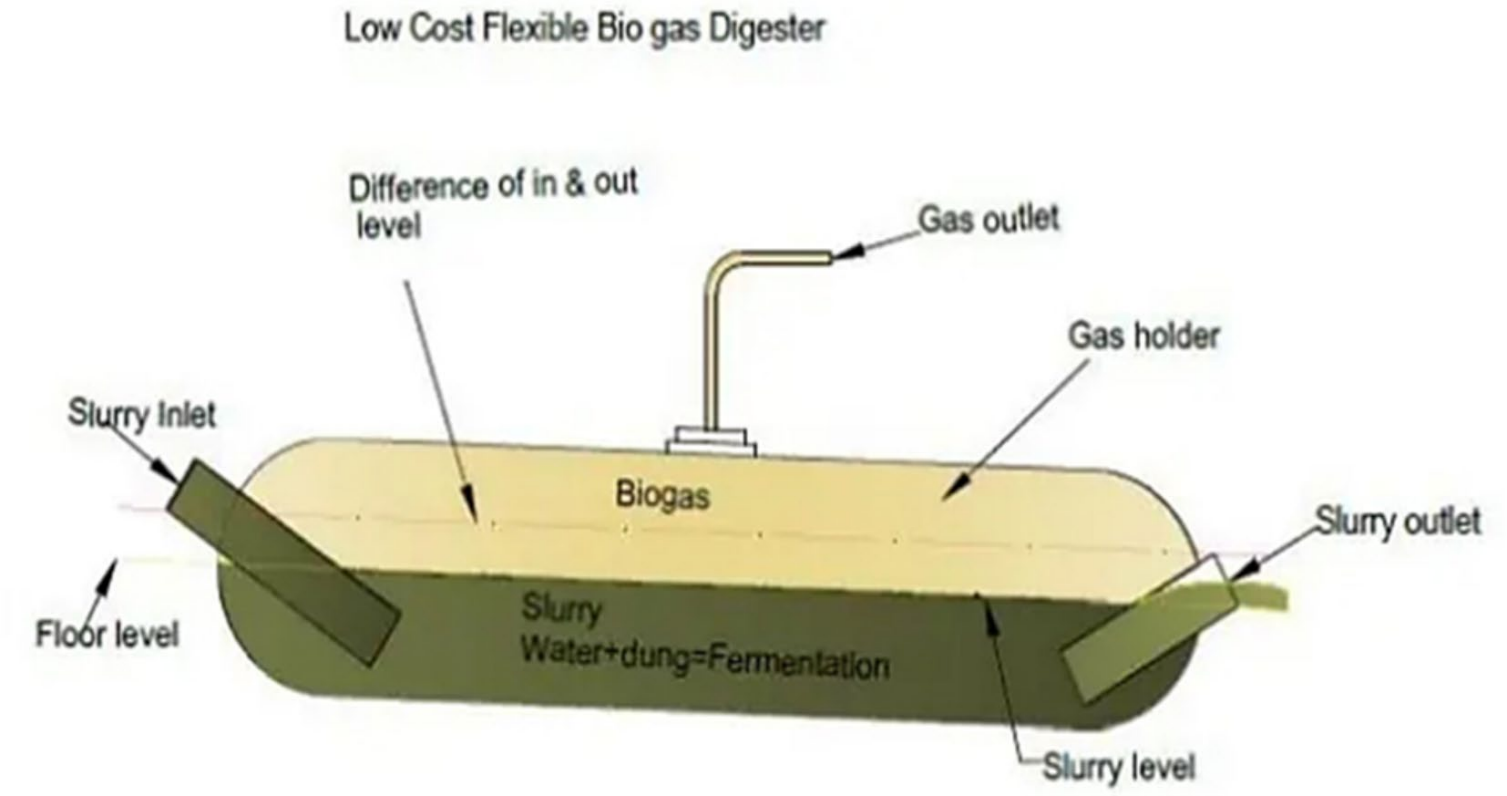
The general concept of the “Balloon or Tube Digester”. Ref.:^43^.

A “Balloon digester”, Fig. 5, comprises a plastic or rubber bag to hold the digester contents. The upper portion is a gas storage space, with the inlet and outlet directly attached to the balloon’s surface. The gas exits the balloon due to internal pressure buildup, which can be augmented by placing weights on it. The movement of the balloon’s surface aids in agitating the fermenting slurry, enhancing digestion. Balloon digesters offer versatility, accommodating even complex feed materials like water hyacinths.

UV resistance is essential for balloon materials, with successful options including (Red Mud Plastic - RMP), Trevira, and butyl. These materials are heat-sealed to form a single unit’s digester and gas holder. However, exceeding the balloon’s pressure limit may damage its surface, necessitating safety valves.

For higher gas pressures, a gas pump may be required. Given the exposure to weather and UV radiation, preference is given to specially stabilized and reinforced plastic or synthetic caoutchouc. Despite these considerations, the useful lifespan of balloon digesters typically ranges from 2 to 5 years^43,44^.

#### Other domestic digester types

**Fig. 6:**
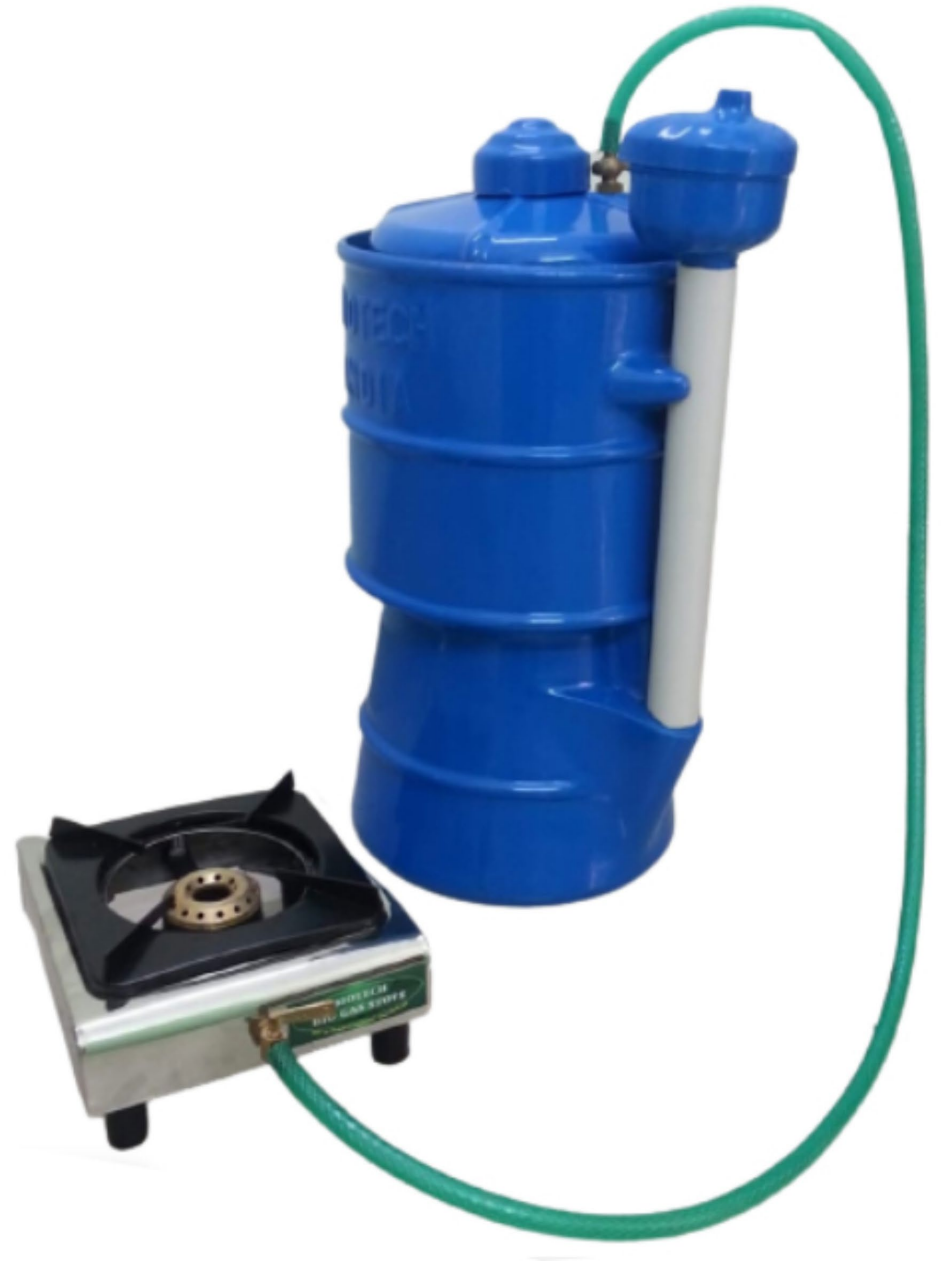
An example of a “Portable biogas domestic digester”. Ref.:^47^.

“Portable home biogas systems”, Fig. 6, are compact, small-scale digesters constructed from either metallic or polyethylene tanks, accompanied by complementary equipment such as gauges, inlets, and outlets. In India, China, and several European nations, numerous companies supply these digesters to the market in various sizes that are customized for installation in domestic environments^45–47^.

Masonry digesters are not necessary for stable soil (E.g., laterite). To prevent seepage, it is sufficient to line the pit with a thin layer of cement (wire mesh fixed to the pit wall and plastered). The pit’s edge is reinforced with a ring of masonry that also serves as anchorage for the gasholder. The gas holder can be made of metal or plastic sheeting. If plastic sheeting is used, it must be attached to a quadratic wooden frame extending into the slurry and anchored in place to counter its buoyancy.

The requisite gas pressure is achieved by placing weights on the gas holder. An overflow point in the peripheral wall serves as the slurry outlet. The Ferro-cement construction type can be applied as a self-supporting shell or an earth-pit lining. The vessel is usually cylindrical. Very small plants (Volume under 6 m3) can be prefabricated. As in the case of a fixed-dome plant, the ferro-cement gasholder requires special sealing measures (proven reliability with cemented-on aluminum foil).

### Research significance

This research investigates the correlation between sustainable architectural design and biogas generation. Its goal is to identify guidelines for integrating architectural layouts with biogas facilities and evaluate current prototypes suitable for implementation in Egyptian households. Additionally, the study discusses architectural limitations and suggests layouts for biogas facilities in various residential structures in Egypt.

## Case study and methodology for Egyptian residential facilities

### Case study

Residential structures in Egypt range widely in style and size, encompassing traditional apartment complexes, residential towers, luxurious villas, mansions, seashore chalets and remote hideaways, quaint village homes, and dwellings found along the southern banks of the Nile River reflecting their unique heritage characteristics.

#### Today’s apartment buildings

**Fig. 7:**
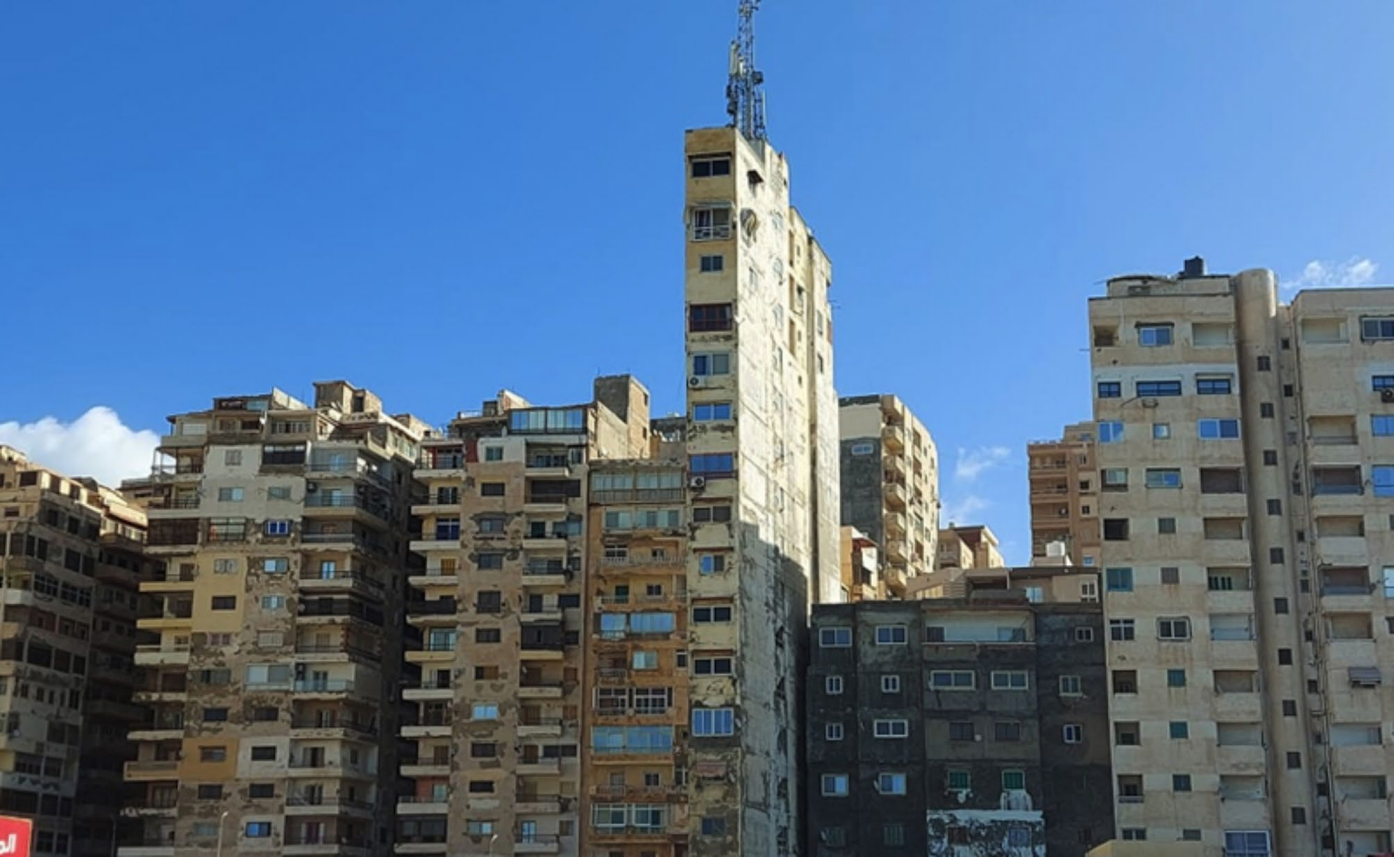
An example of typical Egyptian residential buildings. Ref.:^48^.

In Egypt, what are commonly referred to as residential buildings (Apartment living) are predominantly apartment complexes found throughout various Egyptian cities. Most of today’s Residential buildings in Egypt are built from concrete and bricks; these structures typically consist of multi-story buildings with 2 to 6 apartment units per floor, as shown in Fig. 7.

Additionally, these buildings often feature a basement level for parking and include a small living space for the building porter, usually comprising 1 to 2 bedrooms, a compact kitchenette area, and a small restroom. The building roof contains a single room for storage and access to the elevator’s mechanical control area and the main staircase. Residential buildings in Egypt vary in number of floors, usually from 4 to 6.

#### Condominiums

In the United States and Canada, the term “Condominium” is commonly used to describe residential buildings where the residents own apartment units. A condominium, often shortened to “Condo,” denotes a property ownership arrangement where a building or set of buildings is divided into individual apartment units, either separately or collectively owned, with exclusive occupancy rights by individual owners. These apartment units are usually surrounded by common areas jointly managed by all the apartment unit owners. “Condominium” can refer to the entire building or complex as a whole or individual apartment units. Conversely, in Egypt, most residential buildings have owner-occupied apartments or a mix of rented and owned apartment units.

Theoretically, ownership is the key distinction between a condo and a residential building (Apartment living). However, recently, in the U.S. and Canada, many condominium units have been rented out by individual owners to tenants. Both condominiums and residential buildings in these regions offer similar amenities, such as shared swimming pools, waste disposal services, landscaping, gyms, communal laundry facilities, and parking in building basements. Overall, condominiums are relatively uncommon in Egypt compared to the U.S. and Canada^49,50^.

#### Residential towers

Per Egyptian residential building regulations, residential buildings surpassing nine stories are considered residential towers. This reflects Egypt’s recent contemporary residential development, characterized by spacious, upscale apartments. These towers typically feature basement parking, a living space for the porter, and a rooftop setup similar to conventional residential buildings with fewer floors.

#### Private residencies/villas/mansions

**Fig. 8:**
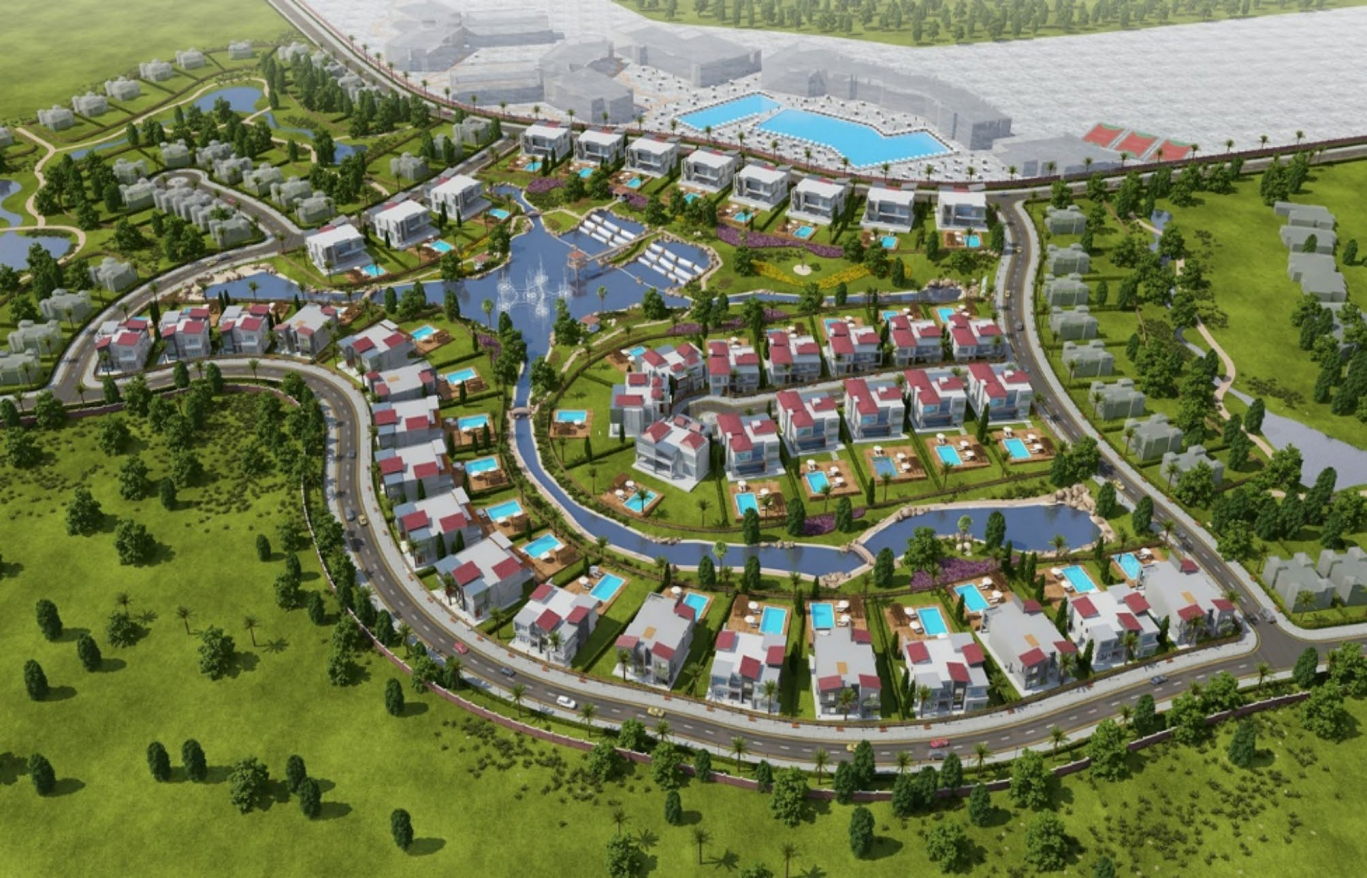
A rendered aerial view of the lake view compound, an example of a luxurious gated community in the 5th settlement district of suburban Cairo in Egypt. Ref.:^51^.

In recent Egyptian real estate, a notable trend is the emergence of expansive gated communities housing luxurious single—and multi-story villas, Fig. 8. These properties feature diverse styles, such as townhouses and mansions, large front and back yard gardens, private swimming pools, dedicated parking spaces, and spacious basements. Villas and mansions are arranged in groups around luxurious lakes and beautiful landscapes.

#### Beach front chalets and remote hideaways

**Fig. 9:**
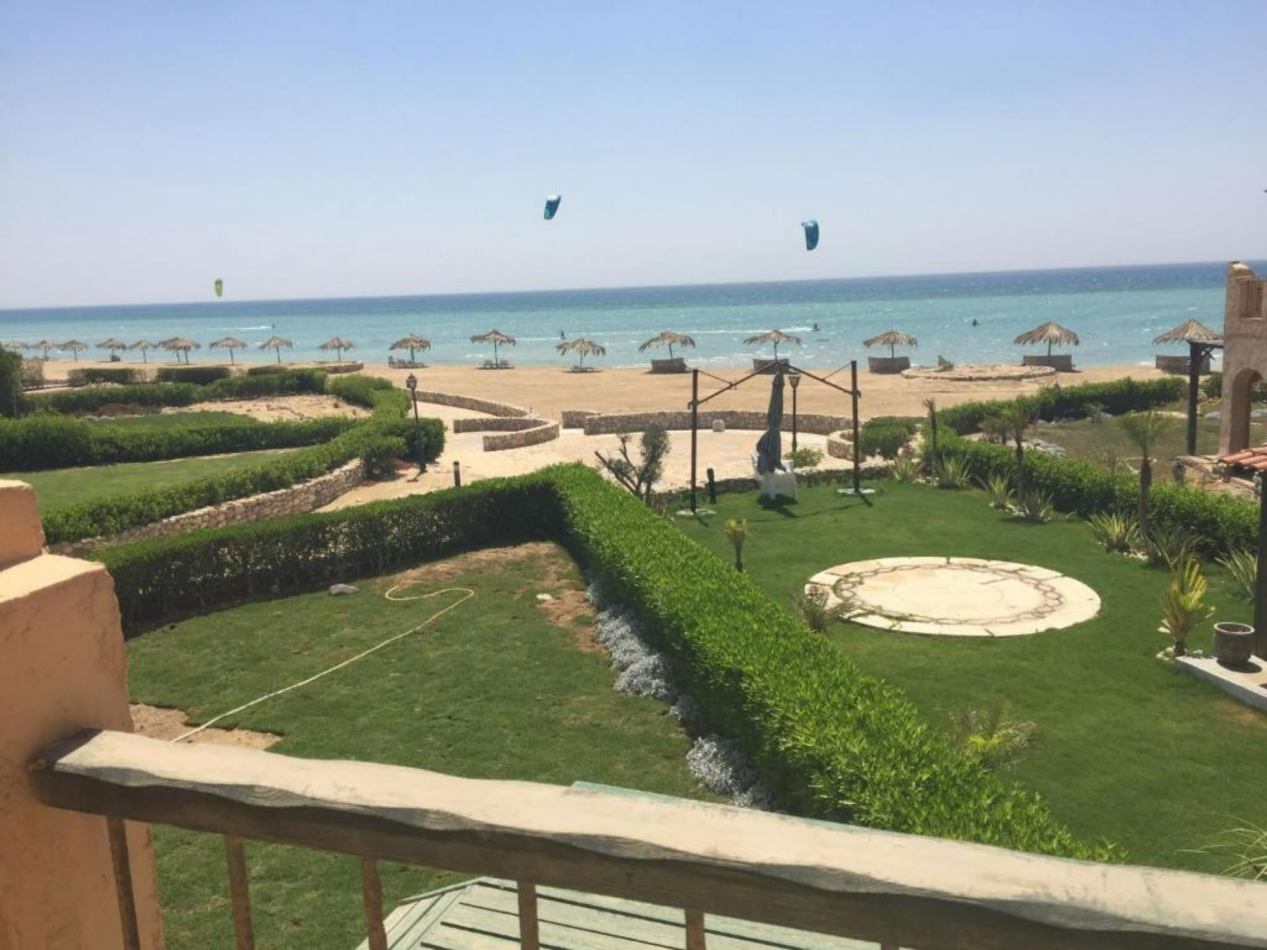
A photograph was taken from a villa’s terrace in the “La Hacienda” seashore compound in Ras Sudr beach in front of Egypt. Ref.:^52^.

Another contemporary real estate trend in Egypt involves establishing gated communities along major Egyptian coastlines featuring chalets and single-story and double-story villas. (Fig. 9) These properties now include front and backyard gardens, with some models even incorporating private swimming pools. The owners of these secluded retreats typically do not reside permanently in them. Instead, they utilize these residential amenities during summer vacations and national holidays.

#### Village/countryside homes

**Fig. 10:**
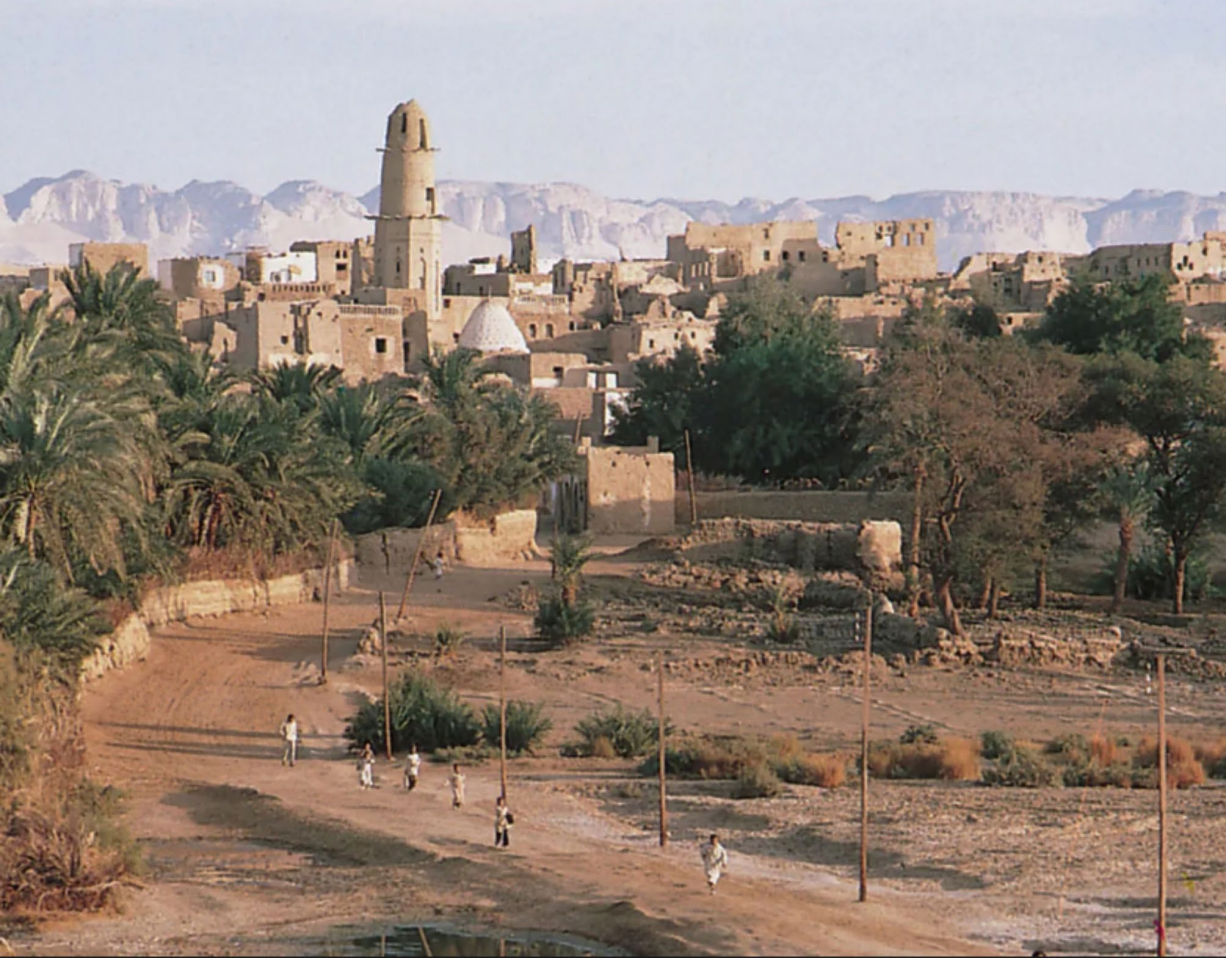
A photograph of a rural Egyptian area with village homes built with mudbricks and limestone. Ref.:^54^.

Village homes in the northern rural areas of Egypt (Fig. 10) showcase the beauty of nature. In these villages, many Egyptians reside in mudbrick houses renowned for their thick walls, which provide insulation against the intense afternoon heat. The flat roofs, exposed to refreshing northern evening breezes, serve as cool sleeping quarters and storage spaces.

Villagers often adorn the outer walls with plaster, typically embellished in blue, a color believed to avert the evil eye. As individuals prosper, they may expand their homes vertically, adding a second story for their married children. Typically, the house’s ground floor comprises guest and living areas, a kitchen, a bathroom, a cattle barn, and harvest storage. These village homes often open to an external courtyard or face the farmer’s land^53^.

**Fig. 11:**
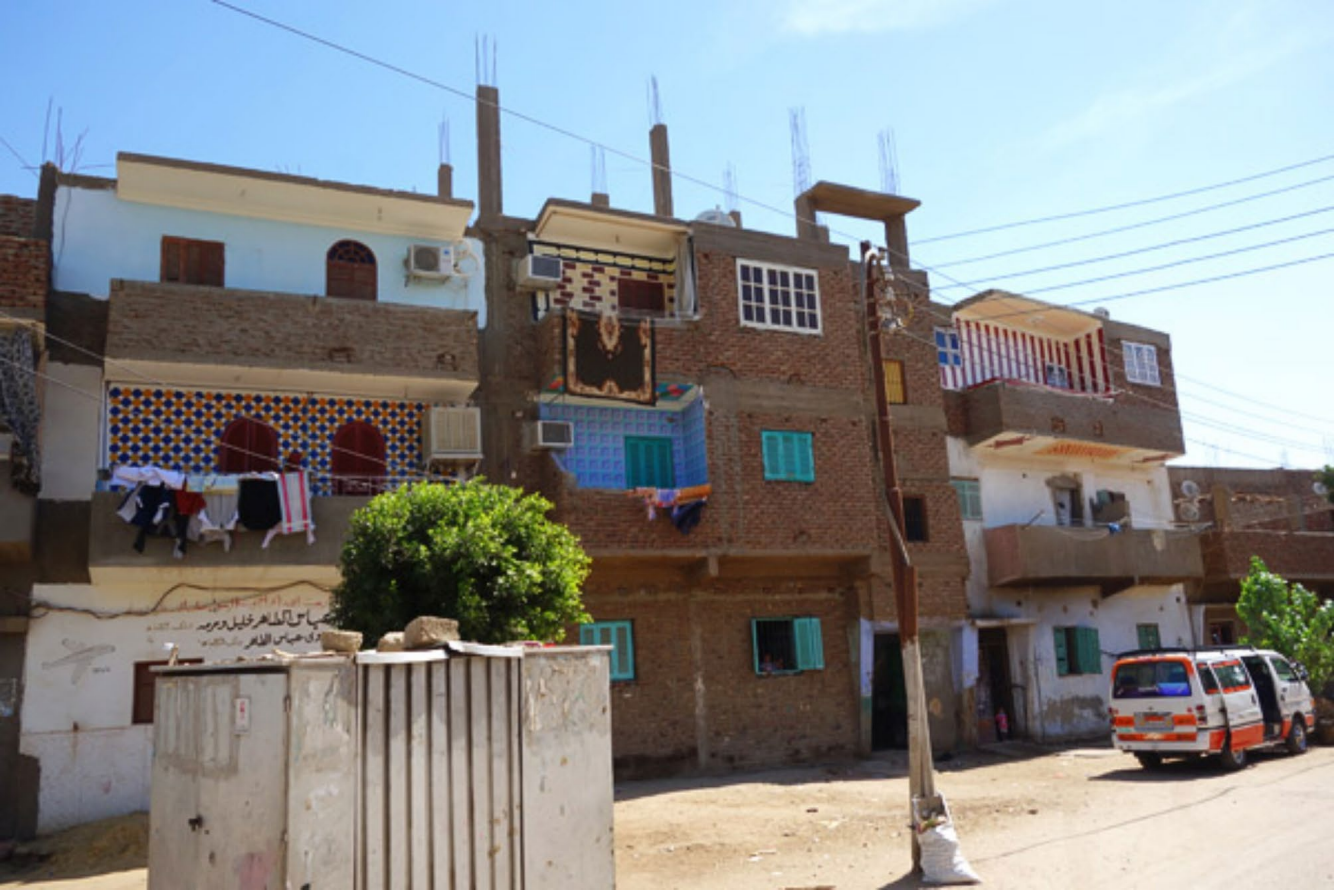
A photograph of a brick and concrete reconstructed Egyptian village home. Ref.:^55^.

In present times, residents of villages have embarked on reconstructing their homes with concrete and red bricks to accommodate additional floors for their sons’ and daughters’ marriages. Figure 11 Despite the new construction’s shift to concrete and bricks, provisions are still made for barns and storing harvested crops within these homes.

**Fig. 12:**
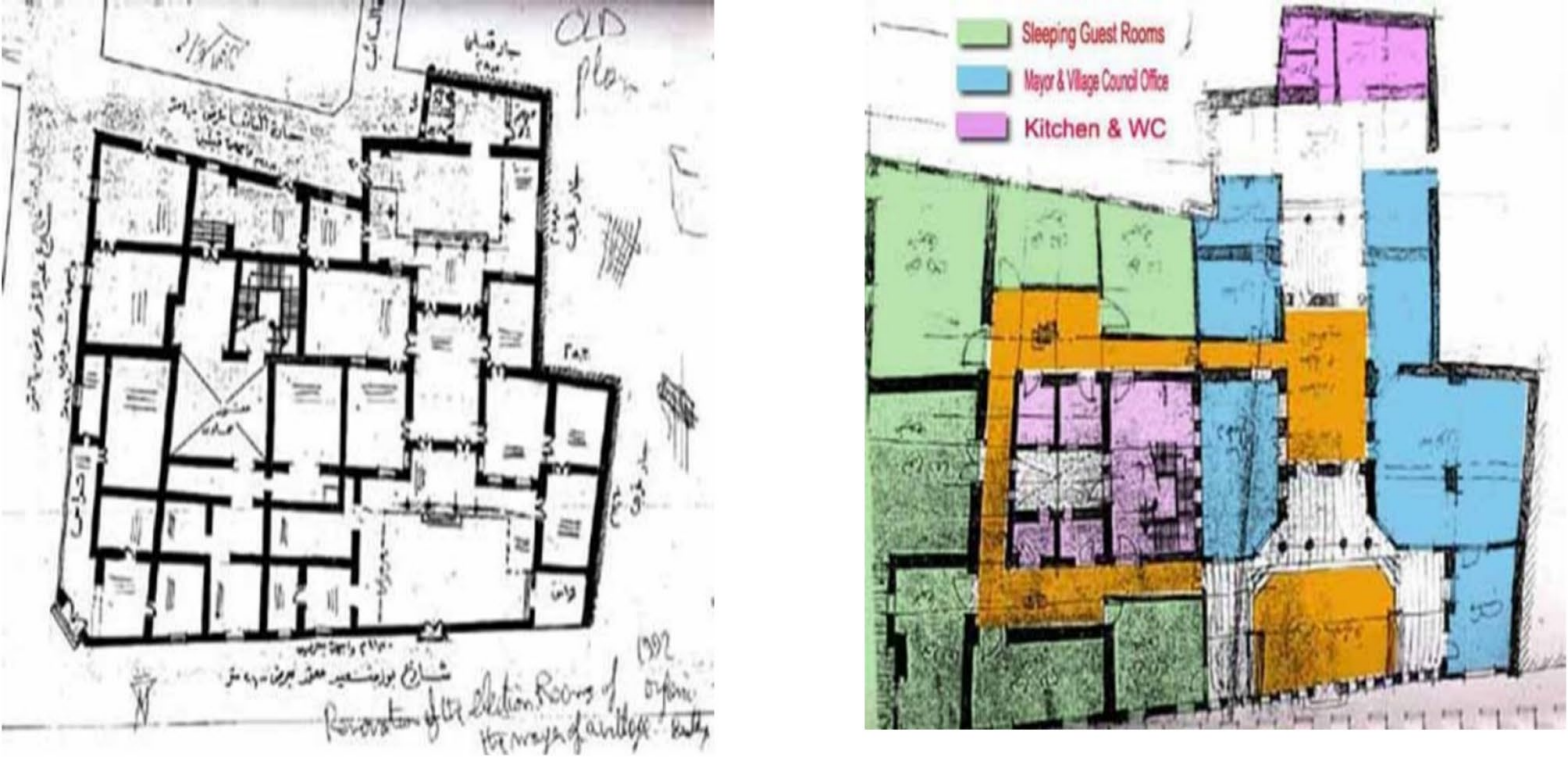
This figure contains an architectural representation of a southern village home of a community leader with guest spaces; the original house is made of mudbrick. Ref.: [Author]

Conversely, in the southern region of Egypt, commonly called Upper Egypt, village homes include an extra area known as the “Madyafa” or a large guest room Fig. 12. In addition to the typical areas found in northern village homes, this space serves as a gathering spot for friends, neighbors, and community members to convene and deliberate on village matters. These “Madyafa” areas are particularly prominent in the residence of the village’s community leader.

#### The Nubian house

The inhabitants of “Nuba” possess an innate architectural acumen honed over generations, enabling them to organically integrate with their surroundings and develop distinctive architectural traits aligned with their environmental, social, and cultural principles.

However, with the construction of the High Dam of Egypt, they were compelled to abandon their ancestral lands and resettle in new government-designated villages in the North. These new settlements featured new housing styles, spatial arrangements, and materials diverging from their traditional vernacular architecture^56–59^.

**Fig. 13:**
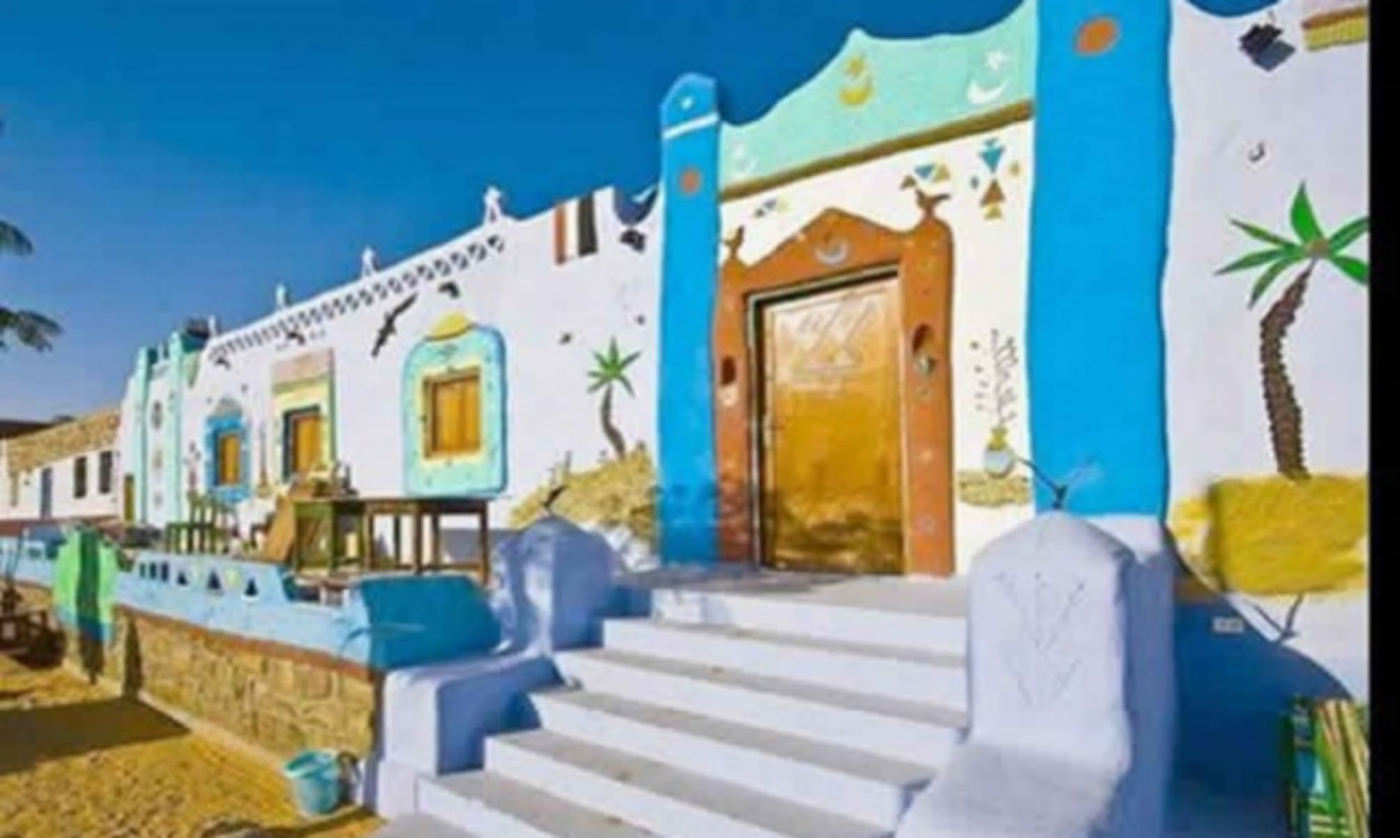
Image of a typical Nubian house. Ref:^57^.

The layouts and orientations differed significantly, and the urban infrastructure failed to fulfill their social norms regarding privacy, familial ties, and community divisions. Figure 13 shows the changes in the style of the Nubian house before and after resettlement.

**Fig. 14:**
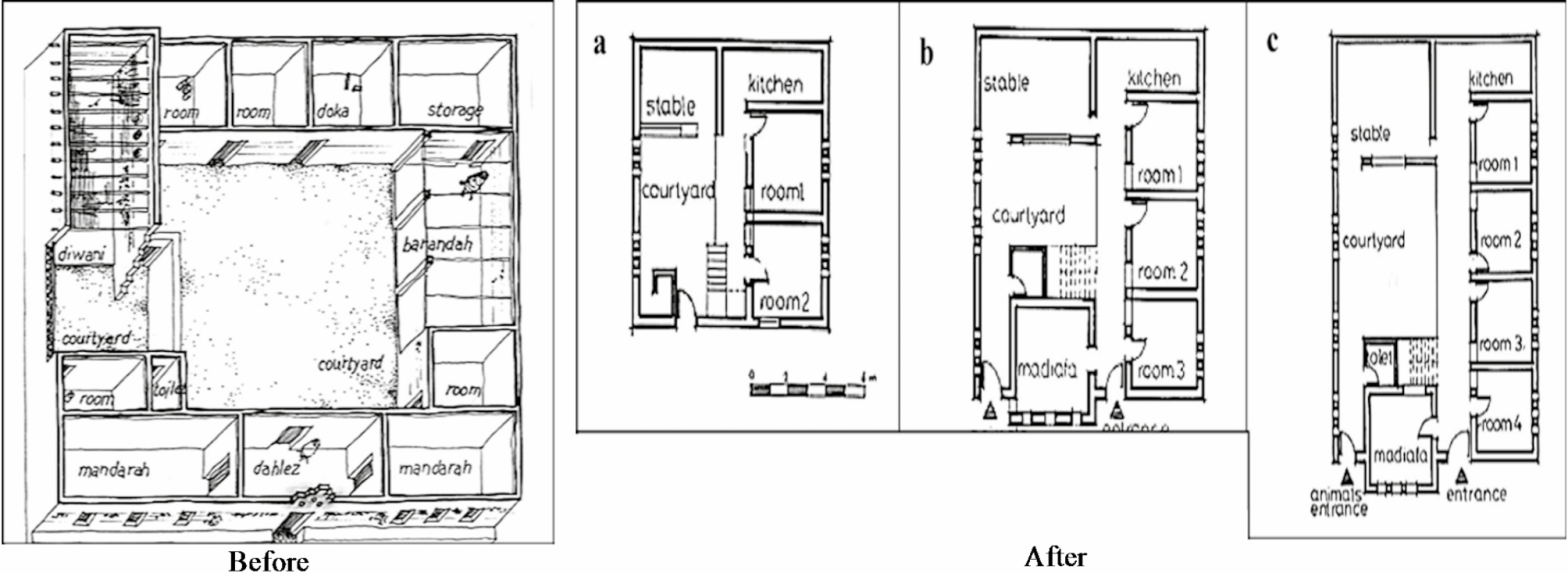
The changes in the style of the Nubian house before and after resettlement. Ref:^59^.

The old Nubian house, Figs. 13 and 14 consists of:

- The entrance gate, “Bawaba” in Arabic, is a 1.2 by 2.4-meter structure located in the center of a tall mud wall near the Nile. Outside the gate, there is a traditional Nubian oven known as “Duka” (A built-in sitting sofa) and balconies made of mud “Silos” that are about 40 cm high. These silos are used to store grain, dates, and other food items.
- The main entrance portico is the area that connects the outside to the inside of the house.
- The “Mandara” in Arabic is the guest room next to the main entrance. It opens to the house’s exterior and inner courtyard and usually has 2 to 3 windows on each side. These windows are the only ones that face the outside, while the rest of the room’s openings are oriented towards the courtyard.
- The courtyard is an open internal space surrounded by rooms on all four sides.
- The kitchen consists of two rooms, usually with a dome-shaped roof and an open vent at the top for ventilation. The middle tray, called “Meshlaa,” serves as a refrigerator for storing food.
- The storage room is a crucial element in the Nubian house.
- The construction materials used in the Nubian house are mud bricks made from mud and small gravel particles.

#### The Siwan house

**Fig. 15:**
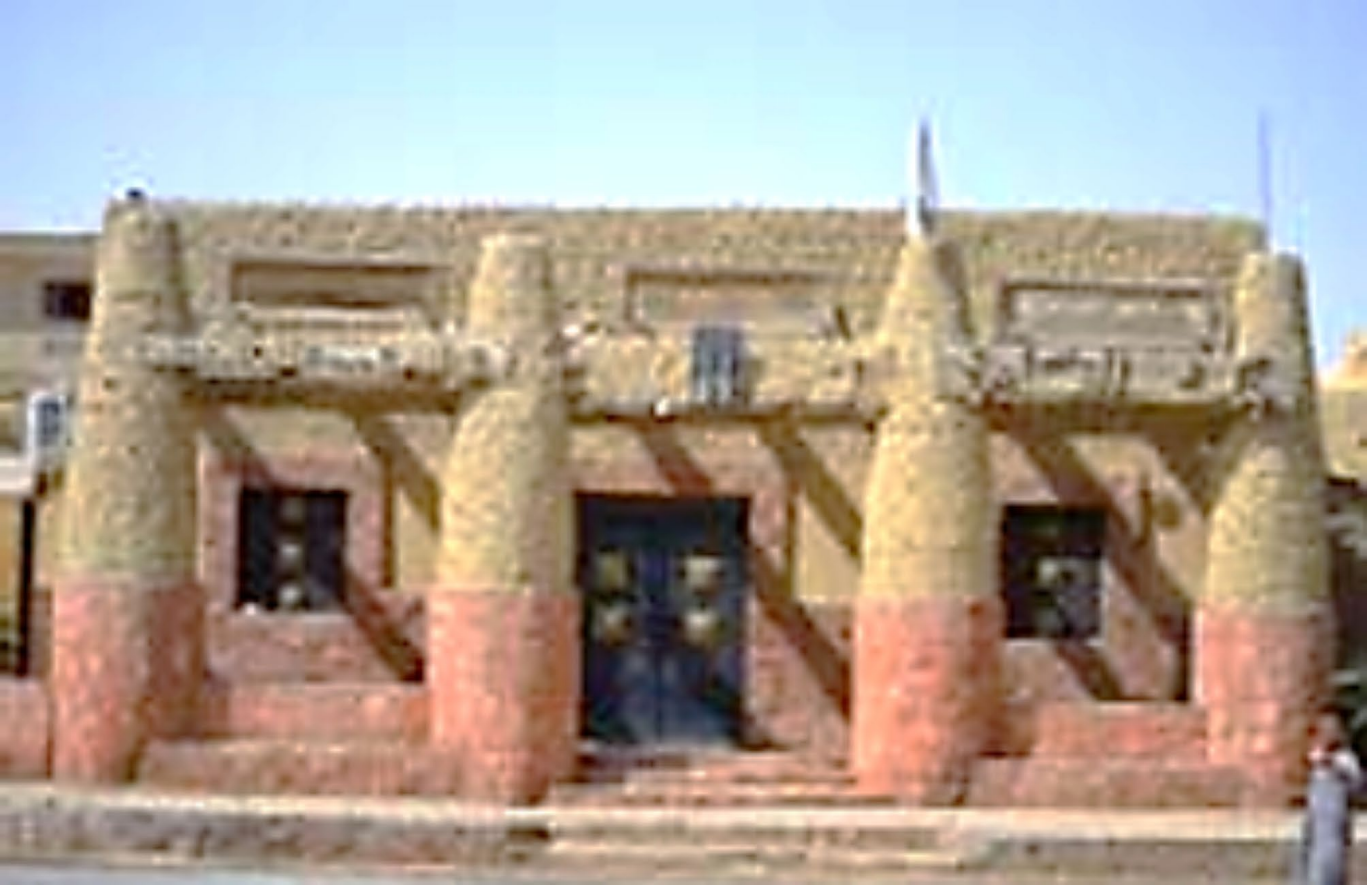
A hospital in Siwa built from Karshif. Ref:^62^.

“Siwa Oasis” is a famous Egyptian tourist destination located between the “Qattara Depression” and the “Great Sand Sea” in the “Western Desert”. It is approximately 50 km (31 miles) east of the Egypt–Libya border and 560 km (350 miles) from Cairo, the capital city. The architecture of the Siwa Oasis is distinguished by using a unique material known as “karshif,” composed of NaCl salt crystals blended with clay and sand impurities, Fig. 15. Blocks of irregular shape are extracted from the salt crust surrounding the saline lake and then cut into smaller blocks for use in masonry. These blocks are joined using a mud mortar rich in salt derived from two different clays, tafla or tiin. Wooden inserts are integrated into the walls to enhance the connection between the external and internal segments, particularly in wider walls.

Traditional Siwan houses are exceptional representations of the Siwa people’s cultural legacy. Constructed from mud bricks, these houses exhibit a distinctive architecture tailored to the hot and arid climate of the region. Key features include flat roofs, spacious internal courtyards, and intricate geometric patterns. Adorned with elaborate mud reliefs and vibrant colors, these houses are typically one or two stories high, with a central courtyard serving as an open court family gathering area. The roofing is crafted from palm fronds supported by wooden beams, while the walls are embellished with intricate mud or straw designs. The compact shape of these dwellings minimizes exposure to direct sunlight, while shaded and tunneled streets protect pedestrians. These streets aid in air circulation and filter sand particles, particularly during sandstorms. The built environment can be categorized as vernacular architecture, showcasing old traditional houses. The minimal number of windows opening onto sidewalks is a deliberate design to prevent adverse climatic conditions from infiltrating indoor spaces^60–63^.

#### The story of “basaysa”

“Salah Arafa” is a university professor and a prominent figure in social entrepreneurship in Egypt and the Arab World; in 1970, he launched an initiative to turn the village of Basaysa into the first sustainable village in Egypt and the Arab world. People in Basaysa are typical farmers. Arafa’s initiative and the Basaysa project were dedicated to empowering the residents of El Basaysa by enhancing their skills. Salah Arafa devised an “Integrated approach to development.”^64^.

His inclusive program encompassed various training sessions covering agriculture, effective natural resource utilization, literacy, teamwork, innovative thinking, and community cohesion. As the scientific advisor to the Sharqia Governorate, Arafa also concentrated on technology transfer to foster genuine progress in El Basaysa, a disadvantaged rural area. Rather than imposing changes externally, he involved community members at every stage, aiming for sustainable development. His research focused on leveraging natural resources to establish eco-friendly rural communities, enhancing residents’ abilities and expertise. A pivotal facet of the El Basaysa initiative involved implementing biogas production units in nearly every household in the village, serving as a renewable energy source. Arafa’s renowned El Basaysa project, launched in the 1970s, has been widely acclaimed as a successful model for sustainable development in Egypt. Within a few years of its inception, Arafa successfully transformed El Basaysa from an impoverished and marginalized village into an eco-conscious community for sustainable development, self-sufficient in biogas production independent of the local gas supply grid. The El Basaysa project showcased Egypt’s early adoption of biogas facilities on a village/communal scale long before the United Nations established the 17 Sustainable Development Goals (SDGs) as part of its global 2030 agenda.

**Fig. 16:**
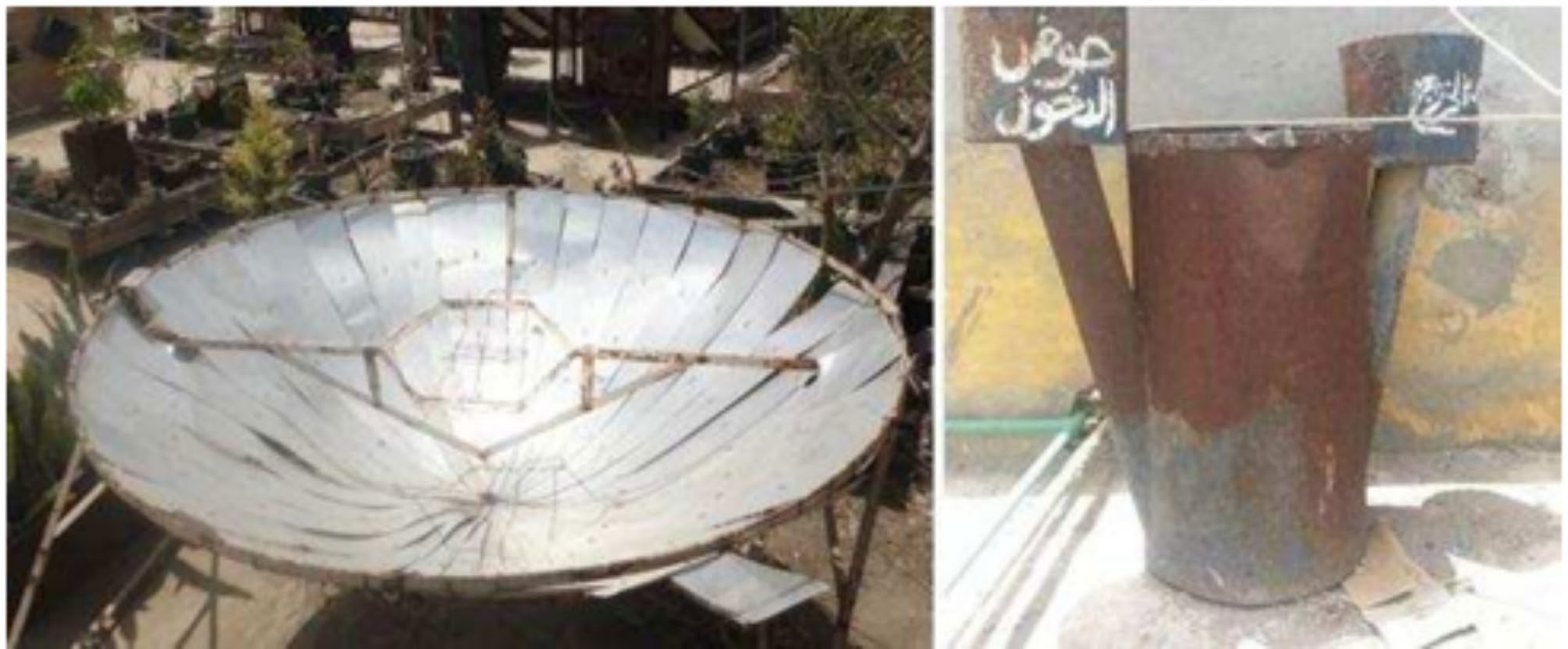
Two Macquets of a solar kitchen (left) and a domestic biogas compartment (right) in Basaysa. Ref:^67^.

Unfortunately, the notable “Basaysa” project has seemingly disappeared very recently, and for unknown reasons, leaving no evidence of its domestic biogas initiatives for documentation or memory. It’s as if nothing remains, not even remnants, except for some few photographs found with difficulty over the internet like those shown in the following, Fig. 16^65–68^.

####  Another Basaysa-Like project in Sinai

**Fig. 17:**
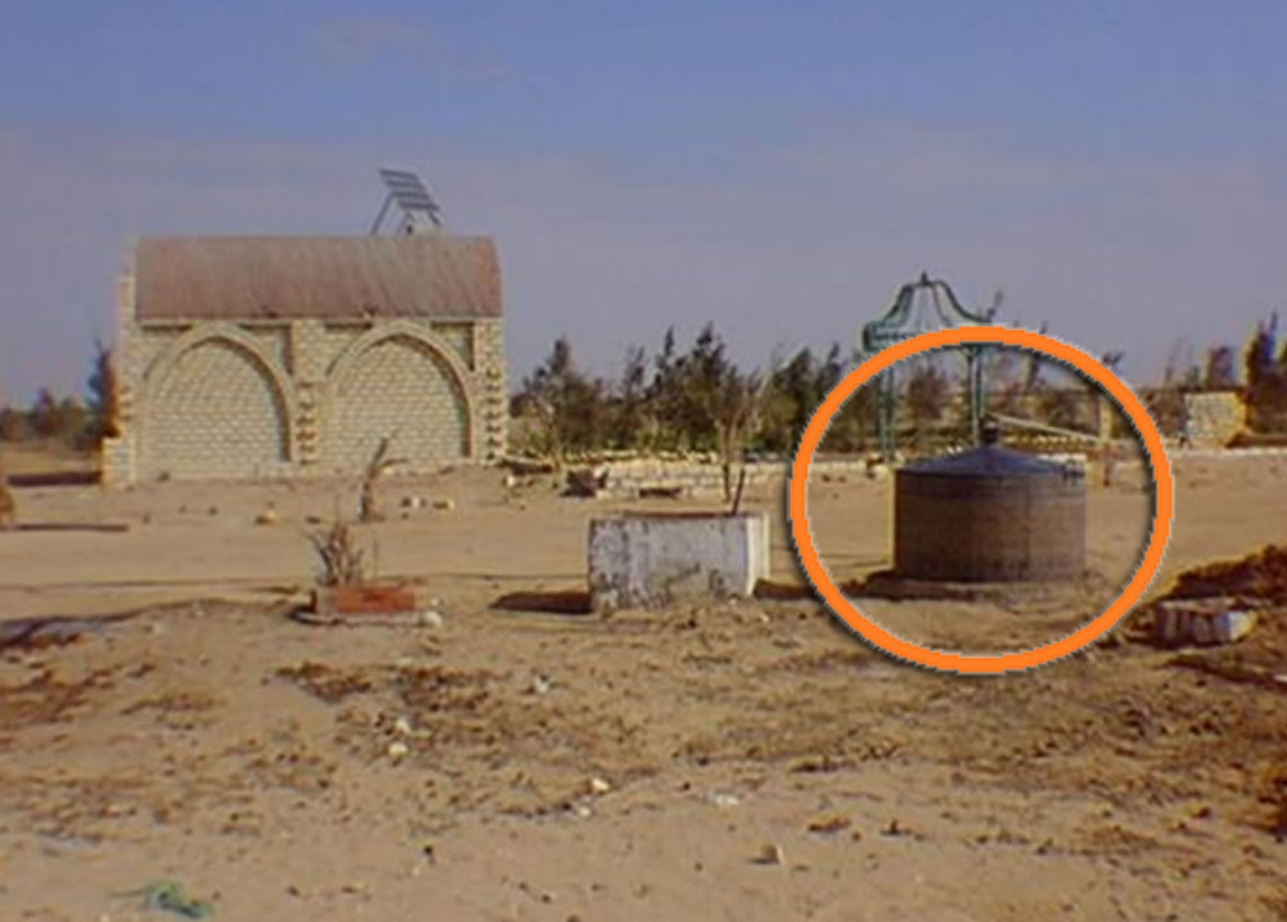
A biogas plant in new Basaysa. Ref:^70^, While the red circle is added to the image by the [Author].

A recently developed Basaysa community in Ras-Sudr, South Sinai, Fig. 17, represents a revival of Professor Salah Arafa’s initial Basaysa project to foster a sustainable community. This Sinai community mirrors Professor Salah’s program, emphasizing the creation of renewable energy sources such as biogas plants^69,70^.

#### “Hayaa Karima” project

**Fig. 18:**
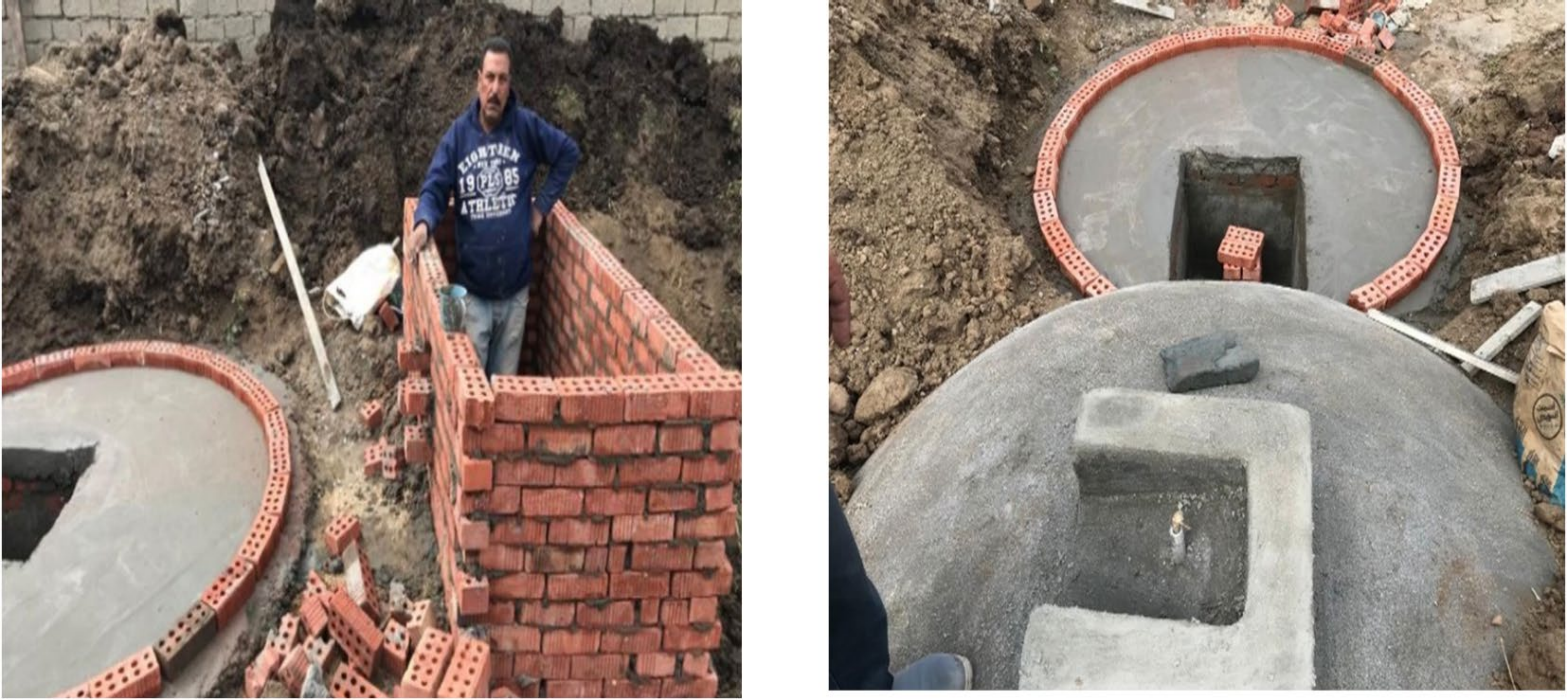
Images of biogas plant in Sunbat of Gharbiya Gov. Ref:^71^.

The “Haya Karima” presidential development initiative has introduced a pivotal project in Egypt’s rural areas, specifically targeting the Gharbiya governorate. The Biogas project initiative aims to generate natural gas and fertilizers for local farmers and livestock farms. Launched in the village of Sunbat in Zefta city within Gharbia governorate, a village under the umbrella of Haya Karima, Fig. 18.

This Biogas project collaborates with the Good Life Foundation, the International Cooperation Fund, and the Renewable Energy Foundation. The primary objective is efficiently utilizing livestock waste to produce natural gas and organic fertilizers. Around 20 units have been successfully established in Sunbat Village as part of this initiative^71^.

#### Other biogas projects in Egypt

**Fig. 19:**
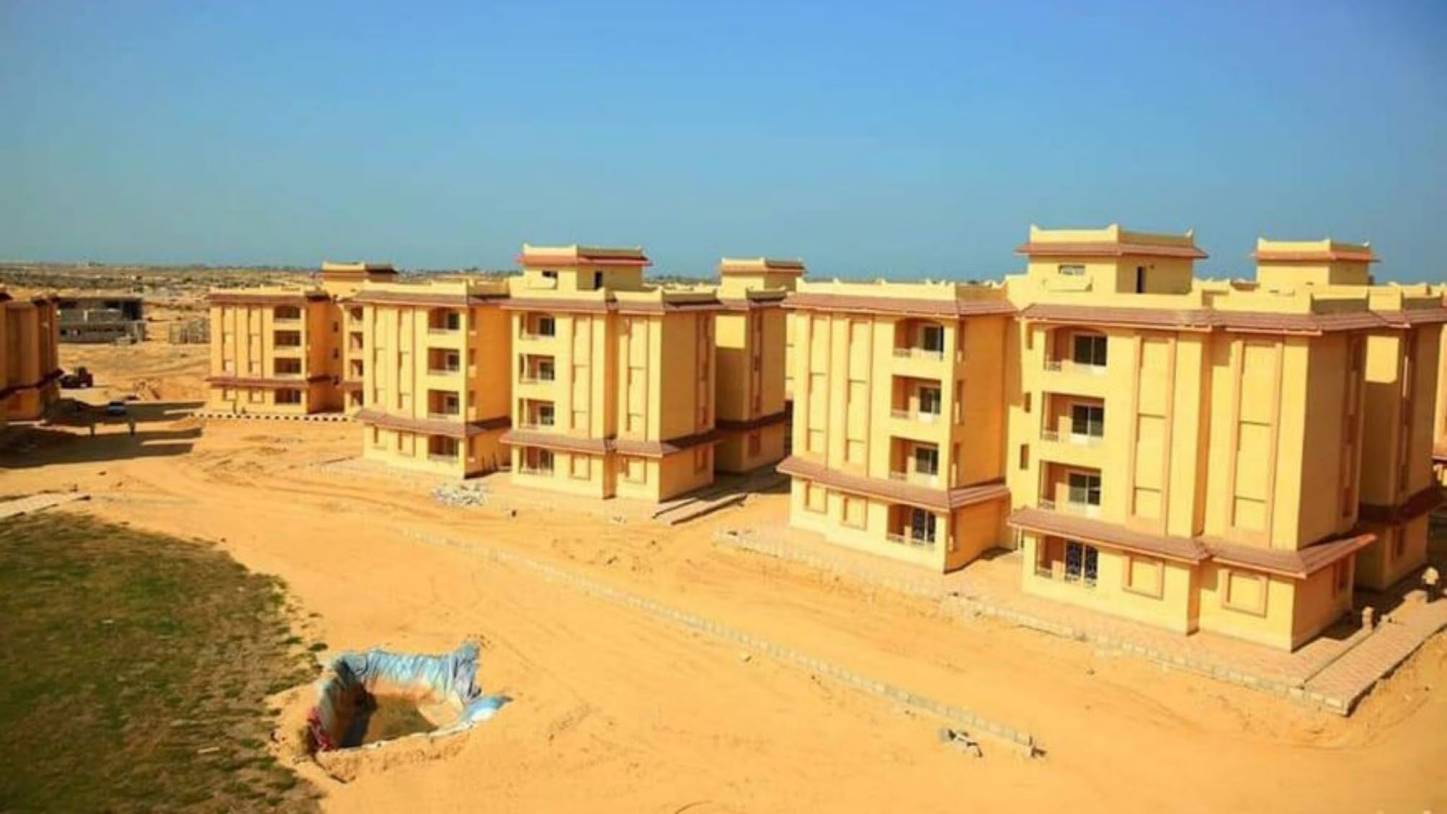
A group of typical Egyptian residential buildings with standard roof spatial configuration. Ref:^77^.

The biogas renewable energy project is also expanding to several other regions in Egypt, such as^72^:

- Assiut Governorate Biogas Plant, Owner: Bioenergy Association for Sustainable Development (BASD, Egypt), Capacity: Not specified, Location: Assiut, Status: BASD, in collaboration with the National Bank of Kuwait, signed an MoU in February 2023. The project aims to establish a centralized biogas plant and compression station to serve 150 beneficiaries across the governorate.
- Fayoum Governorate E3i Bio-Refinery, Owner: Energy3 International (E3i, USA), Capacity: Not specified, Location: Fayoum, Status: E3i partnered with Egypt’s Ministry of Environment, signing an MoU in May 2022. The American company’s project focuses on converting solid waste into biofuel, hydrogen, and graphene for local industries.
- Giza Governorate Waste-to-Energy Plant, Partners: Re-energy Egypt, National Authority for Military Production, Capacity: 30 MW, Location: Abu Rawash, Status: In April 2023, the Re-energy consortium and Giza Governorate sealed a deal for a USD 130 million plant. This plant is designed to convert 1,200 tons of household solid waste daily into electricity. The consortium will construct, own, and manage the plant for 25 years before transferring ownership to the governorate (Fig. 19).

### Methodology and design considerations

#### Technical calculations

Some suggested technical calculations regarding the proposed prototypes:

##### Space utilization

**Fig. 20:**
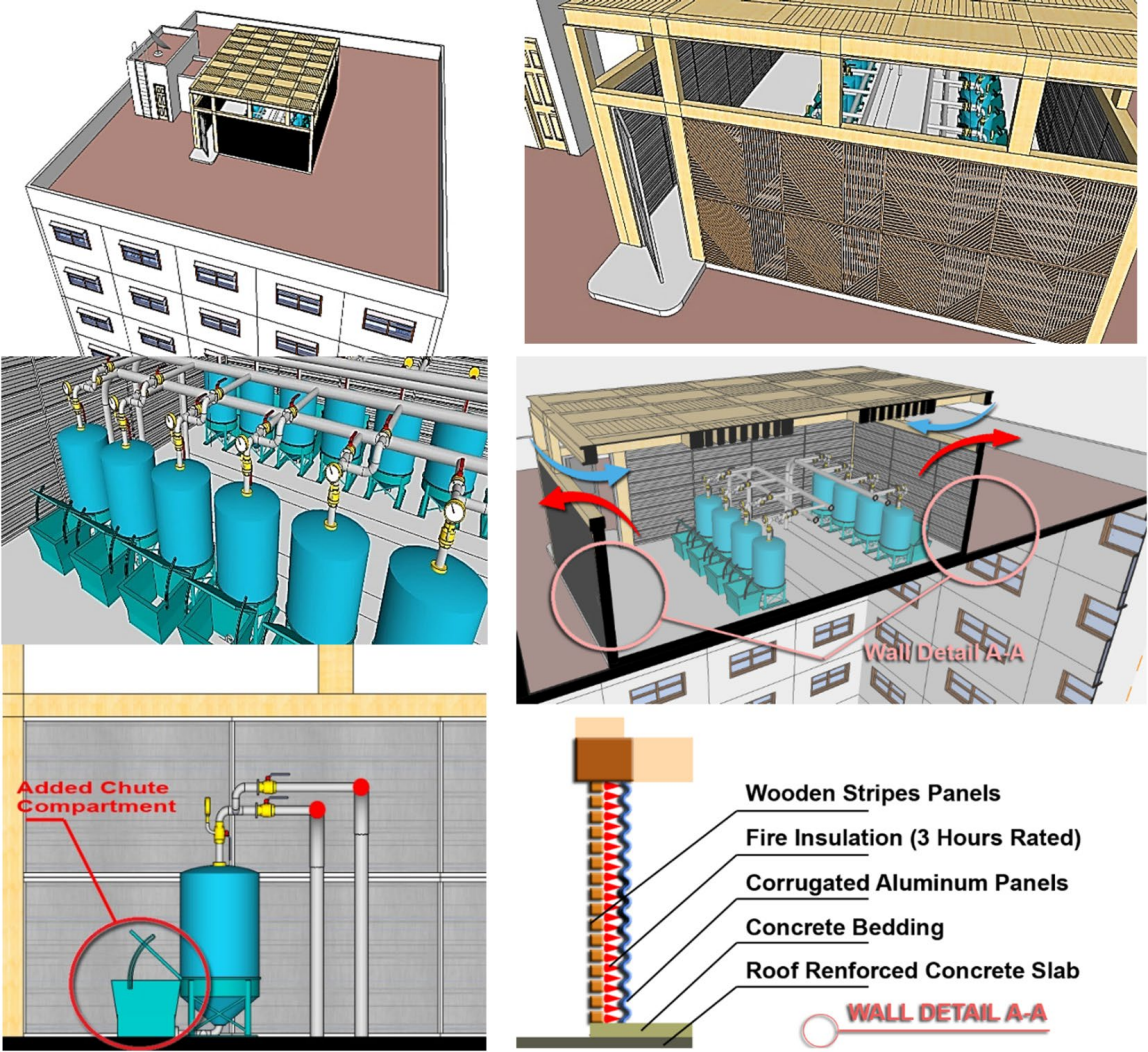
Explains the proposed architectural setup of a lightweight structure acting as a biogas roof plant compartment. Ref. [Author]

Overall, biogas digesters proposed for all residential building types in this research may be classified into three main categories: portable, fixed, and underground biogas digester chambers:

1. Portable Biogas Digesters: These are compact, lightweight cylinders (like the ones shown in Fig. 6), typically made from PVC or polyethylene, making them ideal for installation on the rooftops of apartment buildings. They can be grouped to increase biogas production (Fig. 20).
2. Buried Digesters: These are more suited for villas and mansions, as they can be installed underground in garden areas in front or back yards. This helps optimize space while maintaining the aesthetic appearance of the property and its surroundings.
3. Chamber-Based Digesters: These are larger digester silos placed inside underground chambers similar to street sanitary manholes but with larger dimensions. They are suitable for urban areas and can be installed beneath pedestrian paths, city streets, or public parks/gardens, effectively utilizing underground space.

##### Approximate calculations

**Fig. 21:**
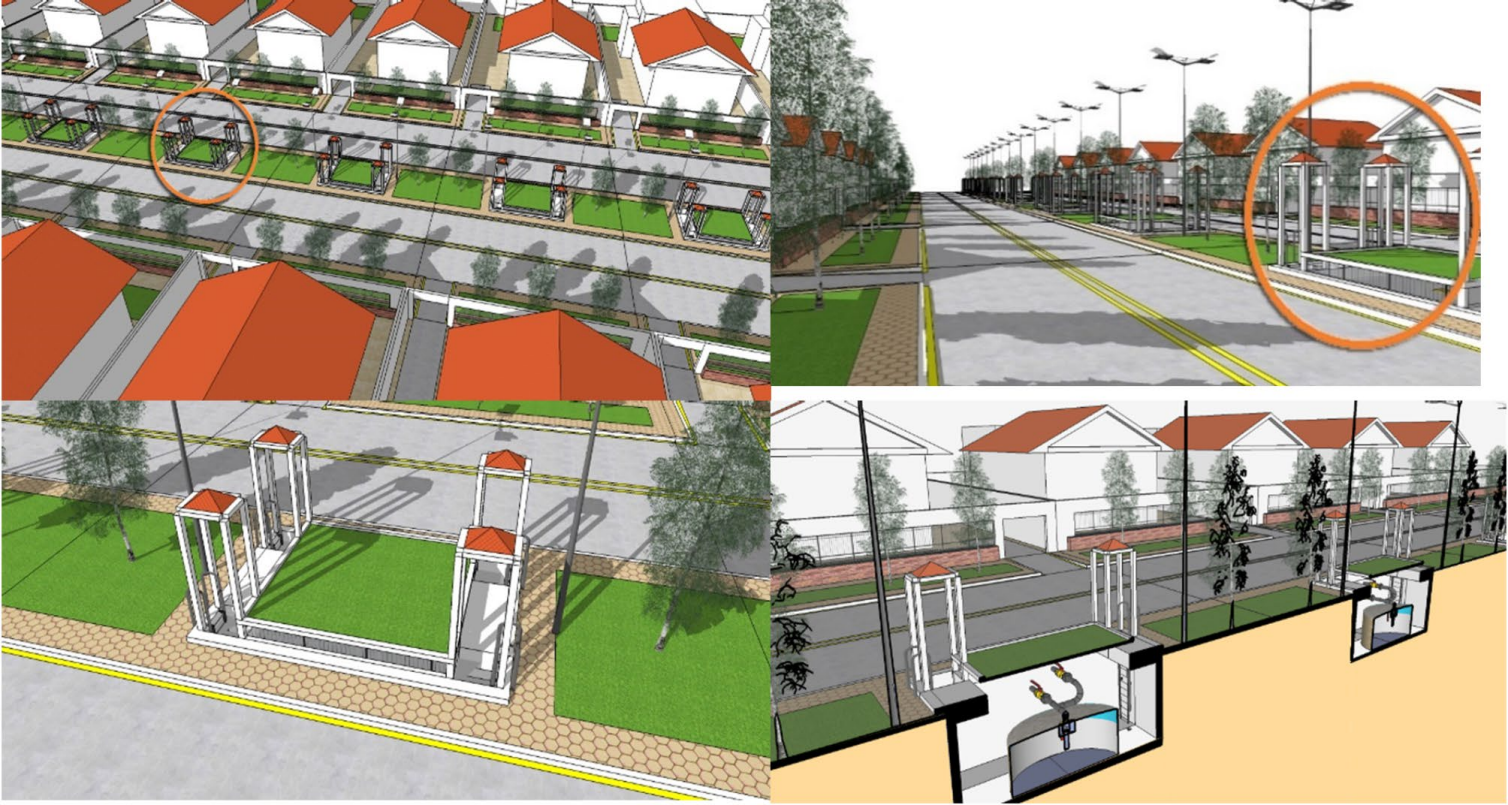
Explains the proposed architectural setup for the installation of the fixed dome digester units for private residencies, villas, and Egyptian mansions. Ref. [Author]

1. For Portable Biogas Digesters placed on residential rooftops: Considering the footprint of a single portable PVC/Polyethylene cylinder digester as approximately 2 m^2^ (this area includes half the width of the passage between it and the adjacent unit in the next aisle, usually known as “Work area” among architects), then the maximum “gross” number of portable biogas digesters that can be placed on a building’s rooftop can be determined by dividing the total rooftop area by the footprint (or “work area”) of one digester. Taking into consideration that these portable digesters are lightweight, thus placing the maximum number of units on the roof may be possible without the need for re-assessing the roof’s load-bearing capacity; for example, a rooftop with a gross area of 100 m^2^ could support up to 50 portable gas units (100 m^2^ / 2 m^2^ = 50 gross biogas units).
2. For Buried Digesters, which are suitable for villas or houses with front and back yards, there are no standardized dimensions for the digester pit in the garden. The size is generally left to the homeowner’s preference. Additionally, no specific code in recent residential building regulations worldwide defines the exact dimensions or depth for fixed digesters in villa yards or gardens. However, as a general suggestion, a garden pit with a 2.5 to 3 m^2^ capacity could be considered a reasonable proposal.
3. Same situation as that of the garden villas buried digesters applies to the Chamber-Based Digesters that are suitable for urban areas; since there are no available regulatory codes by the city municipalities, then as a general guideline, an underground chamber with a capacity of 5 to 6 m³ could be considered a reasonable suggestion including various required clear access areas surrounding the digester inside the chamber. (as shown in Fig. 21)

##### Load-bearing analysis for rooftop installations

As discussed in the “space utilization” above, Portable Biogas Digesters are lightweight, making them well-suited for installation on apartment building rooftops, and can also be grouped to boost biogas production (as shown in Fig. 20). However, the following calculations are provided as simple and general guidelines only since the results may vary depending on the capacity of different portable biogas cylinder models produced by various manufacturers.

##### Approximate calculations

When filled, the weight of the portable digester cylinder is considered by distributing the total weight across the rooftop area to determine the load per square meter. The weight of one filled PVC/Polyethylene biogas cylinder (as in Fig. 6) is 35 kg. approximately^47^. However, to obtain a calculation result tailored to Egyptian conditions, the weight equivalent to that of a traditional Egyptian butane gas cylinder produced by local Egyptian gas companies^73^ It will be used in this calculation, which is a maximum of 27 kg.

For instance, if a rooftop area of 100 m^2^ accommodates 50 portable biogas cylinders, then the total weight of 50 cylinders would be: 50 $\:\times\:$ 27 kg (weight of one cylinder) = 1,350 kg. The next step is to divide the resulting total weight (1,350 kg) by the 100 m^2^ rooftop area, which gives the weight per square meter. This example would result in 13.5 kg/m^2^, significantly below the maximum load-bearing capacity of 250 to 300 kg/m^2^ that the roof can support. This confirms there is no need to redesign the roof slab to accommodate specific bearing loads.

##### Thermal performance of proposed materials under extreme Egyptian summer conditions

The thermal conductivity of PVC is 0.19 W/m·K for a cylinder with a thickness of PVC layer of 0.01 m and with an internal temperature of 15 °C^74^.

*Approximate Calculations*.

Under extreme Egyptian climate conditions that reaches 40 °C to 42 °C in summer, 0.19 W/m·K $\:\times\:$ 15 °C, then divided by 0.01 m = 285 w/m^2^ approximately.

#### Quantitative analysis

Some quantitative analysis regarding the proposed prototypes:

A sensitivity analysis concerning some variations and parameters across different residential setups.

##### From an environmental perspective

Environmental parameter variations across the proposed residential setups are minimal or almost negligible. This is due to the uniformity of Egypt’s natural and climatic conditions across its regions. For instance, extreme temperatures in Upper Egypt rarely exceed 45 °C, similar to those in Lower Egypt near the northern Mediterranean coast. Additionally, Egypt’s topography is predominantly flat, except a small, narrow mountain range called “Mountain A’taaka,” located between the Nile Delta and the Red Sea zone, which is an uninhabited area. The Sinai Peninsula’s inhabitants live in the flat valley areas, while its mountains are sparsely populated. Therefore, all inhabited regions in Egypt share similar environmental, climatic, and geographic conditions, making variations between proposed residential setups almost negligible.

##### From the perspectives of Building technologies & construction

Houses, private residences, villas, city residential buildings, and other suburban homes are now commonly constructed using reinforced concrete, bricks, and other conventional modern building materials. At the same time, people in Egypt’s countryside, including villages, oases, and Nubia, are rapidly transitioning to rebuilding their homes using these modern materials. This shift is driven by the fact that their old traditional homes built with Tafla, mud, or clay are no longer adequate to accommodate their growing families, including married children. It has become increasingly common for villagers to demolish their old homes and replace them with multi-story reinforced concrete buildings, providing separate flats or apartments for each of their married children.

##### From an architectural and aesthetic perspective

Cultural and aesthetic values are rapidly diminishing across all Egyptian residential building setups. The growing demand for more space and the fast population increase are the primary factors influencing recent Egyptian residential architecture. As a result, there is less emphasis on aesthetics or designing homes that reflect cultural or aesthetic values. The primary concern has shifted to meeting the housing needs of families, particularly for accommodating young married children and startup families within limited budgets. In this context, cultural and architectural values are no longer considered in favor of addressing the basic human need for shelter.

As a result, there is a possibility that sensitivity analysis may not be required, as the above influences appear to be consistent across the country.

##### Input conditions for each prototype

Due to the consistency of the above conditions influencing various Egyptian residential building setups across the country, input conditions for each proposed biogas prototype may also be similar.

##### Simulation comparing the biogas yield under varied environmental conditions

As mentioned, environmental conditions are similar across the country, leading to minimal variations due to these factors. However, noticeable differences in biogas yield may depend on other factors, such as the size of the buried and chamber-based digesters and the amount of food waste pumped into them, which can vary between residential areas. For instance, food waste in rural residential digesters may differ significantly from city-based digesters.

Unfortunately, one of the main challenges of this research is the lack of statistical data on this issue. The concept of installing biogas digesters for residential use in Egypt is still relatively new, and almost no similar experimental prototypes are available either. Moreover, the entire biogas issue is a new concept that falls under a greater umbrella of the UN’s SDG sustainability goals, which is also a new area for the government, local NGOs, and the Egyptian public.

#### Safety concerns

Some safety concerns regarding the proposed prototypes:

##### Gas and other leaked substances (FMEA) model

Table 1 is a suggested, straightforward Failure Mode and Effects (FMEA) model for identifying potential causes of gas and other leaked substances^75^.

**Table 1:** Failure Mode and Effects (FMEA) model for identifying potential causes of gas and other leaked substances. Ref. [Author].

Failure mode	Effect	Cause	Detection method	Mitigation
Gas leak	Explosion, health hazards	Faulty valves, pipe corrosion	Gas detectors, pressure sensors	Regular maintenance, replace faulty components
Water leakage	Structural damage, efficiency loss	Poor insulation, material degradation	Visual inspection, moisture sensors	Use of waterproof materials, regular inspections

##### Temperature-induced pressure build-ups (FMEA) model

The following Table 2 is also a suggested, straightforward Failure Mode and Effects (FMEA) model for identifying causes of potential temperature-induced pressure build-ups for the suggested biogas plants^75^.

**Table 2:** Failure mode and effects (FMEA) model for identifying causes of potential temperature induced pressure build-ups for the suggested biogas plants. Ref. [Author]

Failure mode	Effect	Cause	Detection method	Mitigation
Pressure build-up	Tank rupture	Overheating, blocked valves	Pressure gauges, temperature sensors	Pressure relief valves, temperature control systems

##### Risk assessment matrix or case studies from existing biogas installations that May offer practical and preventive safety strategies tailored to each design

Regarding risk assessment, Table 3 is a rough and approximate matrix designed to identify and prioritize potential hazards based on their likelihood and severity. This matrix^76^ is tailored for biogas systems to help determine the maintenance measures needed to minimize the consequences of potential failures:

**Table 3:** A rough and approximate matrix designed to identify and prioritize potential hazards based on their likelihood and severity. Ref. [Author]

Hazard	Likelihood	Severity	Risk level	Mitigation measures
Fire/explosion	Low	High	High	Fire suppression systems, emergency response plans
Structural failure	Low	High	High	Structural integrity assessments, use of high-quality materials

However,

As there are almost no existing residential biogas installations available in Egypt to serve as case studies for implementing similar practical and preventive safety strategies for each of the proposed biogas systems:

*First*.

For the rooftop biogas proposed prototype: Referring to the wall section details (highlighted in Fig. 20) of the room, the architectural wall treatment represents a practical and preventive safety strategy as illustrated in the composition of its walls that consist of three heat insulation layers:

1. The interior wall covering is made of wooden strips. Wood, a poor conductor of heat, helps prevent heat transfer from the outside to the inside, thus maintaining a stable, cooler temperature within the biogas room compared to the external temperature.
2. Fire insulation, which enhances the wall’s fire resistance, gives it a 3-hour fire rating, and serves as a second thermal insulation barrier, further separating the interior from the room’s exterior.
3. Exterior walls clad with corrugated aluminum panels (or potentially corrugated plastic panels) form a third thermal insulation layer, preventing heat transfer from the exterior to the room’s interior.

*Second*.

For buried digesters located in the front or back yard gardens of houses, Two insulation barriers provide a practical and preventive safety strategy:

1. The first barrier is natural, formed by the surrounding soil filling around the buried digester. The soil acts as an effective heat conductor, helping to dissipate any potential increase in the digester’s temperature into the surrounding earth.
2. The second barrier is the material used for the walls of the buried digester, which is typically made from natural and organic materials such as stone, bricks, mortar, or cement, often finished with interlocking tiles. This is a strong thermal insulation layer, further protecting the digester from temperature fluctuations.

*Third*.

For chamber-based digesters, four insulation barriers provide a practical and preventive safety strategy:

1. The first barrier is natural, created by the surrounding soil filling around the chamber-based digester. The soil is an efficient heat conductor, helping dissipate any potential temperature increase from the digester into the surrounding earth.
2. The second barrier consists of the reinforced concrete retaining walls of the chamber, which houses the digester beneath city streets, pedestrian paths, or public gardens and parks. These walls serve as a robust thermal insulation layer.
3. The third barrier is the maintenance-free passage between the concrete retaining walls of the chamber and the digester’s outer walls (as shown in Fig. 21). This passage, which surrounds the digester body, has ceiling openings that allow for ventilation and fresh air circulation into the chamber, acting as an air curtain thermal barrier.
4. The fourth barrier is the material of the digester walls itself, typically made from cement or stone, which forms an additional insulating layer inside the chamber.

## Analysis and discussion

### Research limitations

There is a significant lack of information on biogas initiatives in Egypt. The government, in particular, does not offer public access to data on the advancements of biogas projects in the country. Moreover, scattered online articles are the only available resource for biogas production endeavors. Even the prominent “Basaysa” project appears to have vanished completely, leaving no trace of its household biogas efforts for recording or recollection. It seems as though nothing is left behind - no remnants or valuable photographic material. In addition, the limited photographs uncovered online, as depicted in Fig. 16, lacked pertinent details for the architect to develop the necessary domestic prototypes. These images merely offered basic visuals without providing any helpful or clarified information from which to extract. Additionally, the scarcity of essential and critical hazards and safety parameters in the biogas literature poses another challenge for architects in formulating precise safety design manuals for incorporating safety measures in designing diverse domestic biogas plants in various Egyptian residential buildings. However, the proposed spatial configurations under the result section introduce safety solutions within their design schemes.

### The necessity to understand the architectural characteristics of Egyptian residential buildings concerning the design of the biogas plant prototype

This understanding is important for the following points:

- Adaptation to the Local Context: By understanding the architectural characteristics of Egyptian residential buildings, the domestic biogas plant prototype design can be suitable and compatible with the existing architectural styles and structures in Egypt. This ensures that the biogas production system can seamlessly integrate into residential buildings without compromising aesthetics or functionality.
- Design Customization: Different residential building types in Egypt may have specific architectural features and layouts. By studying these characteristics, it became possible to customize the design of the biogas plant prototype to suit the specific requirements and constraints of each type of building. This ensures that the biogas production system can be efficiently integrated into different residential settings, maximizing its effectiveness and usability.
- Practicality and Feasibility: Understanding Egyptian residential buildings’ architectural characteristics helps assess the practicality and feasibility of implementing biogas production systems in these buildings. Factors such as available space, structural considerations, and building codes can significantly impact the implementation of the biogas plant. Considering these architectural characteristics makes it possible to ensure that the proposed designs are practical and feasible for implementation in real-world residential settings.
- Cultural and Social Acceptance: Architecture plays a significant role in reflecting cultural values and social norms. By studying the architectural characteristics of Egyptian residential buildings, it is possible to ensure that the biogas plant prototype’s design aligns with Egypt’s cultural and social context. This promotes acceptance and adoption of the biogas production system among residents and stakeholders, enhancing its long-term sustainability and impact.
- Adaptation to Building Materials: Analyzing Egyptian building materials leads to customizing the biogas plant prototype design to prevent any adverse effects on the materials and minimize long-term deterioration.
- Space Optimization: Given the limited space in Egyptian residential areas due to the rapidly growing population surpassing 120 million, designing the biogas plant prototype considers unique spatial constraints to utilize available space within buildings efficiently, ensuring seamless integration into the residential environment without excessive space requirements.
- Structural Alignment: Tailoring the design of the biogas plant prototype to match the specific structural considerations of various Egyptian residential buildings, including load-bearing capacities, foundations, and building codes, guarantees structural safety and preserves the integrity of the residential structures.
- Aesthetic Integration: Understanding the architectural styles and visual elements prevalent in Egyptian residential buildings enables the biogas plant prototype design to blend harmoniously with existing structures, ensuring visual coherence and acceptance within the architectural landscape.
- Cultural and Social Sensitivity: By acknowledging the cultural values and social norms embedded in Egyptian architecture, the design of the biogas plant prototype can resonate with the local culture and society, fostering acceptance and integration within the community.

Overall, exploring the architectural characteristics of different Egyptian residential buildings helps design a domestic biogas plant prototype that is contextually appropriate, customized, practical, and culturally accepted.

### Constraints of Egyptian residential buildings to optimize the design of a domestic biogas plant prototype

The constraints are summarized as follows:

- Size and footprint: Egyptian residential buildings often have limited space for additional installations. By understanding the spatial constraints, the biogas plant prototype can be designed to be compact and have a small footprint. This ensures the plant can fit within the available space without significantly modifying the existing buildings.
- Location and placement: Spatial configurations of residential buildings can affect the optimal location and placement of the biogas plant prototype. By understanding the layout of the buildings, the plant can be strategically positioned to minimize disruption to daily activities, maximize accessibility for maintenance and operation, and optimize the flow of materials and energy within the system.
- Integration with existing structures: Egyptian residential buildings often have unique architectural features, such as courtyards, balconies, or rooftop terraces. By understanding these spatial configurations, the biogas plant prototype can be designed to integrate with these existing structures. For example, the plant can utilize the available vertical space or the rooftop area to install solar panels or gas storage tanks.
- Functional optimization: Understanding the spatial configurations of buildings can help optimize the functional aspects of the biogas plant prototype. For example, the location of kitchen or bathroom areas within the residential buildings can influence the placement of biogas outlets or gas pipes. By considering these spatial configurations, the design can ensure that the biogas plant provides convenient access to gas supply for the relevant areas of the buildings.
- Accessibility and safety: Spatial configurations can impact the accessibility and safety of the biogas plant prototype. Understanding the layout of residential buildings can help determine the best access points for the plant’s maintenance, operation, and monitoring. It can also inform safety considerations, such as placing gas detectors or emergency shut-off systems in easily accessible locations.

### Proposed biogas plant Spatial configurations for various Egyptian residential Building types

####  For today’s apartment buildings

The most appropriate digester type for modern apartment buildings is the “Portable biogas domestic digester,” as depicted in Fig. 6. Its suitability stems from the material composition of the biogas silo, typically PVC or polyethylene, akin to water tanks frequently mounted on the roofs of many Egyptian residential buildings. Additionally, polyethylene or PVC silos are available in various sizes and configurations, allowing for increased biogas production when deployed collectively.

Proposed Architectural Spatial Configuration: As previously mentioned, a typical Egyptian residential structure features a basement designated for parking residents’ vehicles and a compact living area for the building caretaker. The roof typically houses a single small room, around 3 m by 3 m minimum, for storage and a small space utilized for elevator mechanical purposes that is usually accessible through the building staircase, Fig. 19. Egyptian building regulations for residential structures prohibit the construction of additional spaces on roofs made of solid materials like brick or concrete. However, it is permissible to add extra spaces on the rooftop using only lightweight materials, such as wood or aluminum panels for walls and asbestos or other lightweight options for the roofing.

While due to safety concerns, it is advisable not to install biogas plants, whether silos or ground-buried, in the building’s basement. Placing them there could result in direct structural damage to the building’s foundation and columns in the event of a plant explosion. To mitigate such risks, a safer alternative is to position the biogas plant on the roof. This placement ensures that any released gases can disperse freely into the open atmosphere in the event of a leak or explosion. However, the primary critical consideration when installing the biogas plant on the building’s roof is the exposure to intense sunlight and high temperatures during Egypt’s summer, which can soar to 40 to 45 degrees Celsius. This exposure can rapidly increase the biogas plant’s internal temperature, causing the gas inside to expand and explode. The illustration in Fig. 20 details a suggested architectural configuration involving a lightweight structure constructed from wood and aluminum panels intended to serve as a compartment for a biogas roof plant.

- Typically, Egyptian apartment buildings have footprints ranging between 20 and 25 to 30 m and 20 to 25 to 30 m. This implies ample rooftop space is available on these buildings to accommodate a lightweight structure for a biogas plant.
- The designated space features a wooden pergola that can vary in size based on the number of “Portable biogas domestic digesters” installed. The upper part of the walls remains open to allow external ventilation, serving to release excess summer heat, functioning like architectural “Badjirs” (Wind Catchers).
- The walls consist of an external wooden striped panel, an internal sandwiched fire insulation (rated for 3 h), and corrugated aluminum sheets facing the biogas units on the inside.
- Each portable digester can have a chute box or compartment for direct food waste filling.
- Portable digesters can be placed individually or in clusters with a shared piping system. Each apartment has its digester, while all units are connected via a single piping network to maximize biogas production.

#### For condominiums

As previously stated, the idea of condominiums is not as prevalent in Egypt as it is in the United States and Canada. However, should condominiums be present in Egypt, the architectural configuration suggested for the apartment buildings above would suit Egyptian condominiums. Both condominiums and apartment buildings adhere to the same building codes and regulations in Egypt. The only major distinction lies in the ownership of the people living in these types of buildings rather than architectural requirements; condominiums do not necessitate a distinct biogas plant setup from that suggested for apartment buildings.

#### For residential towers

The same situation applies to Egyptian residential towers, which share the same building codes and regulations for roof settings as apartment buildings and condominiums.

#### For private residencies/villas/mansions

In Egypt, the most suitable biogas unit for private residences, villas, and mansions is the “Fixed Dome Digester.” These residences are located in upscale communities where the main attraction is their front and back gardens. Owners of such properties value these houses for their outdoor garden spaces and often purchase them for activities such as playing, barbecuing, swimming in pools, and enjoying pleasant summer evenings under the open sky. Consequently, residents of these luxury homes do not prefer or accept the idea of installing biogas units in their garden areas, not to mention the potential hazards of these biogas units. To address this challenge, the proposed solution involves the installation of fixed dome digester units in a collaborative network arrangement within the curb of the road island facing the residential units. Referencing Fig. 21:

- This recommended architectural design entails establishing below-ground manhole-like chambers at equally spaced intervals on the street island, accessible via vertical ladders. The chamber’s walls are constructed from concrete with a fire rating of 3 h, a concrete floor, four vertical stair access points situated at each corner of the chamber, and a ceiling height elevated 80 centimeters above pedestrian/curb level to facilitate ventilation through upper windows akin to a wind catcher.
- The chamber’s roof can be cultivated with grass or vegetation to contribute to the overall street greenery.
- In this concept, the fixed dome digester is not submerged in the ground but rather placed in this chamber with ample surrounding space from the chamber walls to enable maintenance access.
- The chamber is also outfitted with firefighting equipment.

#### For beach front chalets and remote hideaways

**Fig. 22:**
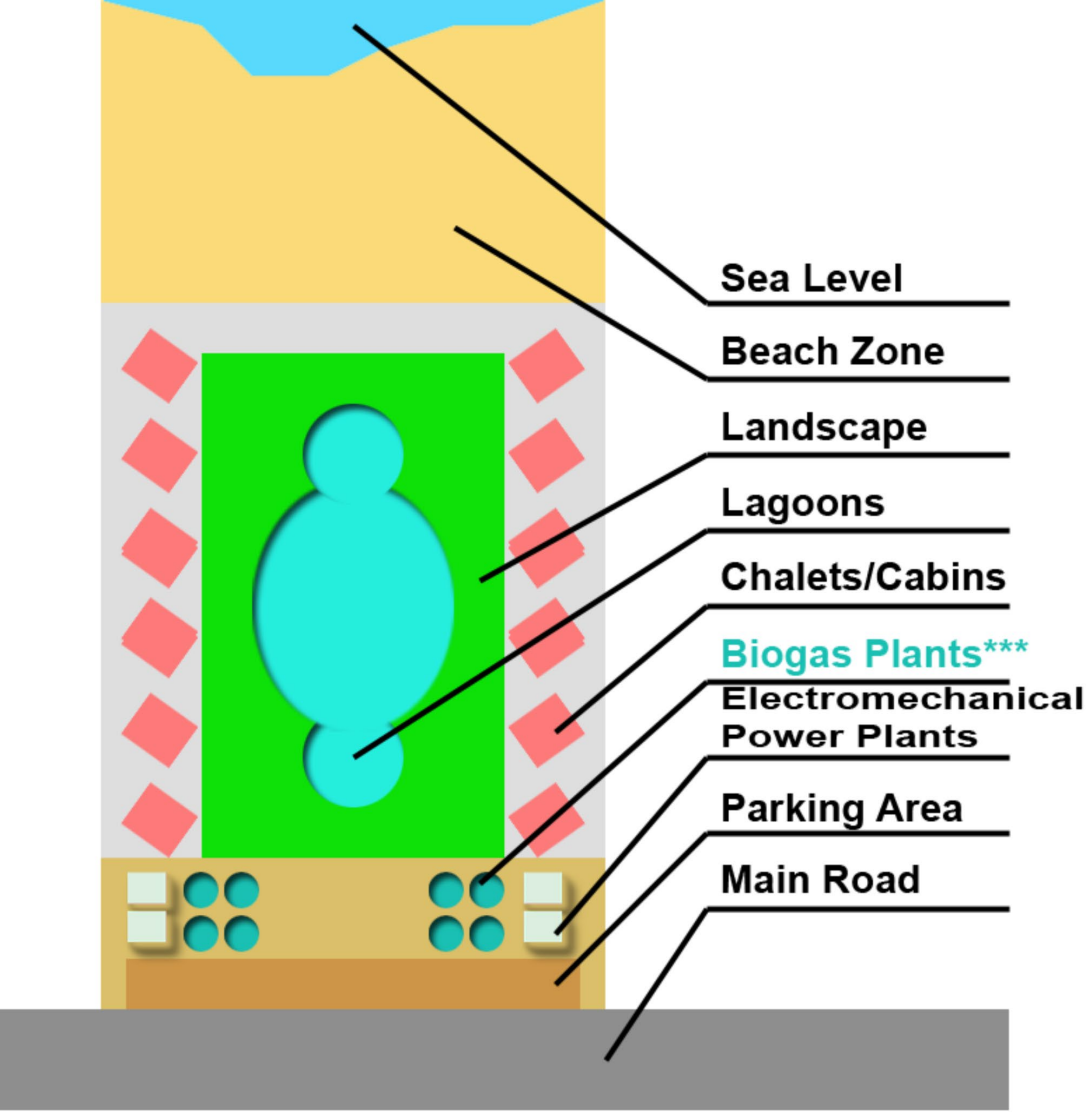
A schematic diagram showing the architectural components and spatial zoning of a typical Egyptian tourist village; the schematic also shows the best zone for the proposed biogas plant. Ref. [Author]

In Egypt, beachfront chalets and secluded retreats are commonly found within enclosed communities known as “Tourist villages”. These villages typically feature clustered chalets and cabins of varying sizes, along with picturesque landscaping elements like grass, vegetation, lagoons, and children’s play areas. Security within these gated communities is typically overseen by private security firms. According to the Egyptian building regulations governing tourist villages, no structures—whether made of concrete, bricks, or even lightweight materials—are permitted to be built on the beach area. Additionally, any form of underground chambers or excavation is strictly prohibited in the beach zone. Most Egyptian lands designated for tourist village real estate are positioned so that the beach area is at the front, while the rear faces a highway or a high-speed road, with the village entrance opening onto it. A typical master plan for a tourist village consists of grouped chalets, cabins, or villas arranged from the beachfront extending towards the property’s rear, ensuring unobstructed views of the sea. In the central area of the village, green spaces, landscaping, lagoons, and various recreational features are situated, while other amenities such as power plants, water reservoirs, security and administrative facilities, and parking lots are in the back of the land near the main entrance of the village. as shown in the following (Fig. 22):

For optimal positioning, biogas plants should be placed in the amenities zone located at the back of the village, as illustrated in the figure above. The ideal type of biogas digester for tourist villages is the “Fixed Dome Digester,” which can be buried directly in the soil without needing specific architectural configurations, except for grouping these digesters in clusters interconnected by a piping system.

####  For village and countryside homes

Given the limited space for constructing new residential structures and the high urban densities within the Egyptian village, it becomes challenging to allocate additional areas between residential buildings for the installation of biogas plants. In such cases, the optimal suggestion is to position biogas plants at the outer edges of the urban zone, akin to that adopted in the Ras Sudr New Basaysa project in Sinai. where “Fixed dome digesters” is placed at specified distances outside the urban or village community area, as depicted in its provided figure. There are no architectural settings required for installing these digester types as it can be buried directly in the soil, except for grouping these digesters in clusters interconnected by a piping system.

#### For the Nubian house

In a traditional Nubian house featuring an internal open court area, the house owner typically allows the installation of a biogas plant anywhere within this courtyard. In such instances, a fixed dome digester, Floating Cover Digester, or a Balloon/Tube Digester is deemed suitable for these traditional houses. Since, Privacy is paramount for Nubian house owners, who prefer to exclusively own their biogas plant without sharing the produced biogas with neighbors. Installing any of these three common types does not necessitate any special architectural adjustments.

Conversely, in modern Nubian houses where the traditional internal courtyard is absent from the architectural layout, a biogas plant setup akin to those in the Ras Sudr New Basaysa project in Sinai would be more appropriate.

#### For the Siwan house

The biogas proposal designed for Nubian homes can also be applied to Siwan homes due to potential similarities in traditional home architectural layouts, cultural perspectives, and the current transitions towards a more modern housing design layout.

### Challenges in scaling the proposed design(s)

Some challenges for scaling the proposed design(s) prototypes:

#### Developing modular design frameworks and suggesting standardized components that can be adapted for varying capacities

As mentioned earlier, due to the lack of statistical data and similar residential biogas plant experiments in Egypt and due to the novelty of the concept to the government, NGOs, and local municipalities, it is not yet possible to finalize a modular design with standardized components that can be adapted for varying capacities. Since biogas production is still in its early stages, further development of this concept will subject to future research.

### Field validation and/or pilot studies regarding the proposed prototypes

Some field validation and/or pilot Studies for the proposed prototypes:

*Providing empirical data from pilot installations in representative Egyptian residential settings*.

As mentioned earlier, due to the lack of statistical data and similar residential biogas plant experiments in Egypt and due to the novelty of the concept to the government, NGOs, and local municipalities, providing empirical data represented one of the major challenges that faced this research.

#### Referencing comparable successful projects in similar Climatic and cultural conditions

As mentioned, there are almost no existing residential biogas installations available in Egypt to be compared with successful projects in similar climatic and cultural conditions,

However, the following experiment could serve as a successful project in regions with similar climatic and cultural conditions^78^: A group of researchers from the United Arab Emirates (UAE) and Lebanon developed a hybrid photovoltaic (PV) and biogas production system for residential use.

This system addresses Lebanon’s energy crisis, particularly after several megawatts of off-grid PV systems were deployed in the country amidst an economic downturn that impacted the fuel and energy sectors. The hybrid system incorporates a prediction component that forecasts PV generation and calculates the waste demand needed from the biogas system to compensate for PV generation shortfalls.

Using machine-learning techniques, the system compares PV generation with user demand, switching to biogas when solar power is insufficient. If the available biomass is inadequate, a diesel generator serves as a backup. The system considers various types of manure (cattle, swine, poultry) and food residues in its calculations.

In a simulation based on a real high-capacity manufacturing operation, the system demonstrated the ability to meet energy demands with PV and biogas. For instance, in January, 3.4 tons of beef-type manure generated 143.83 cubic meters of methane, though the system initially underestimated the methane required. Over time, the system’s error rate decreased.

Their study highlighted the importance of the proposed system in improving energy efficiency, particularly in areas with unreliable weather conditions.

### Cost benefit analysis

Some economic issues like cost-benefit Analysis regarding the proposed prototypes:

#### Life-cycle costing (LCC) and energy payback period

What follows is a suggested approach^79^ to provide a quick economic perspective, integrating life-cycle costing (LCC) and energy payback period calculations for the proposed biogas designs compared to traditional energy solutions.

#### Life-cycle costing (LCC)

Life-cycle costing involves assessing all costs associated with a project throughout its entire lifespan, including initial investment, operational, maintenance, and disposal costs. Below is a structured criterion to use:

1. Initial Investment Costs:

- Biogas Plant: Includes costs for constructing the digester, installing gas storage, piping, and other necessary infrastructure.
- Traditional Energy Solutions: Involves costs for setting up infrastructure for natural gas or electricity supply, as well as heating system installations.

2. Operational Costs:

- Biogas Plant: Includes costs for feedstock (organic waste), labor, maintenance, and periodic inspections.
- Traditional Energy Solutions: Covers costs for fuels (natural gas, coal, etc.), electricity, and routine maintenance.

3. Maintenance Costs:

- Biogas Plant: Includes regular upkeep of the digesters, gas storage, and piping systems.
- Traditional Energy Solutions: Involves maintenance of boilers, heaters, and electrical systems.

4. Disposal/End-of-Life Costs:

- Biogas Plant: Involves decommissioning of digesters and disposal of residual waste.
- Traditional Energy Solutions: Includes the decommissioning of infrastructure and disposal of hazardous materials.

5. Environmental Costs:

- Biogas Plant: Potential savings from reduced greenhouse gas emissions and improved waste management.
- Traditional Energy Solutions: Costs related to carbon emissions, pollution control, and environmental degradation.

#### Energy payback period (EPP)

The energy payback period refers to the time needed for an energy system to generate the same amount of energy that was initially invested in its construction and operation.

1. Biogas Plant:

- Energy Input: Energy required for constructing the digester, processing feedstock, and running the plant.
- Energy Output: Energy produced from biogas, such as electricity, heat, or fuel.

2. Traditional Energy Solutions:

- Energy Input: Energy needed for extracting, processing, and transporting fossil fuels, as well as building the necessary infrastructure.
- Energy Output: Energy generated from natural gas, coal, or other conventional energy sources.

#### Example calculations in U$ dollars

1. For a Biogas Plant with an Initial Investment of $100,000, Annual Operational Costs $10,000, and an Annual Energy Output: 500 MWh, the Energy Payback Period will be equal to: the Initial Investment divided by the Annual Energy Output = $100,000 / 500 MWh = 0.2 years.
2. For a Traditional Energy Solution (Natural Gas) with an Initial Investment of $50,000, Annual Operational Costs of $20,000, and Annual Energy Output: 500 MW, the Energy Payback Period will be equal to the: Initial Investment divided by the Annual Energy Output = $50,000 / 500 MWh = 0.1 years.

#### Comparison

1. Cost comparison:

For Biogas: Higher initial investment but lower operational costs and significant environmental benefits.

For Traditional Energy: Lower initial investment but higher operational costs and environmental impact.

2. Energy payback period:

For Biogas: Slightly longer payback period but offers sustainability and waste management benefits.

For Traditional Energy: Shorter payback period but higher long-term environmental costs.

## Conclusions

In general, architects should prioritize understanding the physical and safety aspects of biogas when customizing biogas plant designs for domestic and residential use, as highlighted by the essential biogas production key parameters in the study. A key characteristic of biogas is its notable lighter-than-air property and lower calorific value compared to natural gas. Suggested safety measures addressing both gas and liquid leaks are as follows:

- There is a significant lack of information on biogas initiatives in Egypt.
- For gas leaks, the architect should implement a design with the upper walls of the biogas room open in a “Badjir” style to facilitate the rapid dispersal of any leaked gas.
- For liquid leaks: Architects should carefully supervise biogas plant construction and consider using chemical insulating materials on internal walls to prevent erosion and inhibit fungal and bacterial growth. Collaboration with chemical science experts is recommended to select suitable insulation materials and effectively address this issue.
- This study presents more suitable proposed spatial configurations for biogas plants in various types of residential buildings in Egypt.

## Data Availability

Data is provided within the manuscript.
